# 2015 Brainhack Proceedings

**DOI:** 10.1186/s13742-016-0147-0

**Published:** 2016-11-01

**Authors:** R. Cameron Craddock, Pierre Bellec, Daniel S. Margules, B. Nolan Nichols, Jörg P. Pfannmöller, AmanPreet Badhwar, David Kennedy, Jean-Baptiste Poline, Roberto Toro, Ben Cipollini, Ariel Rokem, Daniel Clark, Krzysztof J. Gorgolewski, R. Cameron Craddock, R. Cameron Craddock, Daniel J. Clark, Samir Das, Cécile Madjar, Ayan Sengupta, Zia Mohades, Sebastien Dery, Weiran Deng, Eric Earl, Damion V. Demeter, Kate Mills, Glad Mihai, Luka Ruzic, Nick Ketz, Andrew Reineberg, Marianne C. Reddan, Anne-Lise Goddings, Javier Gonzalez-Castillo, Krzysztof J. Gorgolewski, Caroline Froehlich, Gil Dekel, Daniel S. Margulies, R. Cameron Craddock, Ben D. Fulcher, Tristan Glatard, Samir Das, Reza Adalat, Natacha Beck, Rémi Bernard, Najmeh Khalili-Mahani, Pierre Rioux, Marc-Étienne Rousseau, Alan C. Evans, Yaroslav O. Halchenko, Matteo Visconti di Oleggio Castello, Raúl Hernández-Pérez, Edgar A. Morales, Laura V. Cuaya, Kaori L. Ito, Sook-Lei Liew, Hans J. Johnson, Erik Kan, Julia Anglin, Michael Borich, Neda Jahanshad, Paul Thompson, Sook-Lei Liew, Daniel S. Margulies, Marcel Falkiewicz, Julia M. Huntenburg, David O’Connor, Daniel J. Clark, Michael P. Milham, R. Cameron Craddock, Ramon Fraga Pereira, Anibal Sólon Heinsfeld, Alexandre Rosa Franco, Augusto Buchweitz, Felipe Meneguzzi, Jörg P. Pfannmöller, Rickson Mesquita, Luis C. T. Herrera, Daniela Dentico, Vanessa Sochat, B. Nolan Nichols, Anibal Sólon Heinsfeld, Alexandre Rosa Franco, Augusto Buchweitz, Felipe Meneguzzi, Julio E. Villalon-Reina, Eleftherios Garyfallidis

**Affiliations:** 1 Computational Neuroimaging Lab, Center for Biomedical Imaging and Neuromodulation, Nathan Kline Institute for Psychiatric Research, Orangeburg, NY USA; 2 Center for the Developing Brain, Child Mind Institute, New York, NY USA; 3 Centre de Recherche, Institut Universitaire de Gériatrie de Montréal, Montréal, Quebec Canada; 4 Department of computer science and operational research, University of Montreal, Montreal, Canada; 5 Max Planck Research Group for Neuroanatomy & Connectivity, Max Planck Institute for Human Cognitive and Brain Sciences, Leipzig, Germany; 6 SRI International, Menlo Park, CA USA; 7 Department of Psychiatry and Behavioral Sciences, Stanford University, Stanford, CA USA; 8 Functional Imaging Unit, Center for Diagnostic Radiology, Unviersity Medicine Greifswald, Greifswald, Greeifswald Germany; 9 Centre de Recherche, Institut Universitaire de Gériatrie de Montréal, Montréal, Quebec Canada; 10 Université de Montréal, Montréal, Quebec Canada; 11 University of Massachusetts Medical School, Worcester, MA USA; 12 University of California, Berkeley, CA USA; 13 Institut Pasteur, Paris, France; 14 University of California, San Diego, La Jolla, CA USA; 15 University of Washington, Seattle, WA USA; 16 Center for the Developing Brain, Child Mind Institute, New York, NY USA; 17 Poldrack Lab, Department of Psychology, Stanford University, Stanford, CA USA; 18 Computational Neuroimaging Lab, Center for Biomedical Imaging and Neuromodulation, Nathan Kline Institute for Psychiatric Research, Orangeburg, NY USA; 19 Computational Neuroimaging Lab, Center for Biomedical Imaging and Neuromodulation, Nathan Kline Institute for Psychiatric Research, Orangeburg, NY USA; 20 Center for the Developing Brain, Child Mind Institute, New York, NY USA; 21 Montréal Neurological Institute, McGill University and Institute of Pscychology, Montréal, Québec Canada; 22 Douglas Mental Health Institute, Montréal, Québec Canada; 23 Otto-von-Guericke University, Magdeburg, Germany; 24 Montreal Neurological Institute, McGill University, Montreal, QC Canada; 25 University of Hawaii John A. Burns School of Medicine, Honolulu, Hawaii USA; 26 Oregon Health & Science University, Portland, OR USA; 27 University of Greifswald, Greifswald, Germany; 28 Duke Institute for Brain Sciences, Durham, NC USA; 29 Department of Psychology and Neuroscience, University of Colorado, Boulder, CO USA; 30 Institute of Cognitive Neuroscience, University College London, London, United Kingdom; 31 Section on Functional Imaging Methods, Laboratory of Brain and Cognition, National Institute of Mental Health, Bethedsa, MD USA; 32 Department of Psychology, Stanford University, Stanford, CA USA; 33 Computational Neuroimaging Lab, Center for Biomedical Imaging and Neuromodulation, Nathan Kline Institute for Psychiatric Research, Orangeburg, NY USA; 34 Center for the Developing Brain, Child Mind Institute, New York, NY USA; 35 City University of New York-Hunter College, New York, NY USA; 36 Max Planck Research Group for Neuroanatomy & Connectivity, Max Planck Institute for Human Cognitive and Brain Sciences, Leipzig, Leipzig Germany; 37 Monash Institute of Cognitive and Clinical Neurosciences, Monash University, Melbourne, Australia; 38 McGill Centre for Integrative Neuroscience (MCIN), Ludmer Centre for Neuroinformatics and Mental Health, Montreal Neurological Institute (MNI), McGill University, Montréal, Québec Canada; 39 University of Lyon, CNRS, INSERM, CREATIS., Villeurbanne, France; 40 Department of Pscyhological & Brain Sciences, Dartmouth College, Hanover, NH USA; 41 Instituto de Neurobiología, Queretaro, Queretaro Mexico; 42 Neural Plasticity and Neurorehabilitation Laboratory, Chan Division of Occupational Science and Occupational Therapy, Division of Biokinesiology and Physical Therapy, Keck School of Medicine Department of Neurology, University of Southern California, Los Angeles, CA USA; 43 Carver College of Medicine, The University of Iowa, Iowa City, IA USA; 44 The Saban Research Institute of Children’s Hospital, Los Angeles, California USA; 45 Department of Pediatrics of the Keck School of Medicine, University of Southern California, Los Angeles, CA USA; 46 Chan Division of Occupational Science and Occupational Therapy, USC, Los Angeles, CA USA; 47 Division of Physical Therapy, Department of Rehabilitation Medicine, Emory University School of Medicine, Atlanta, GA USA; 48 Imaging Genetics Center, Laboratory of Neuro Imaging, Keck School of Medicine of USC, University of Southern California, Los Angeles, CA USA; 49 Chan Division of Occupational Science and Occupational Therapy, Division of Biokinesiology and Physical Therapy, Department of Neurology of the Keck School of Medicine, USC, Los Angeles, CA USA; 50 Max Planck Research Group for Neuroanatomy & Connectivity, Max Planck Institute for Human Cognitive and Brain Sciences, Leipzig, Germany; 51 Center for Biomedical Imaging and Neuromodulation, Nathan Kline Institute for Psychiatric Research, Orangeburg, NY USA; 52 Center for the Developing Brain, Child Mind Institute, New York, NY USA; 53 PUCRS, Porto Alegre, Rio Grande do Sul Brazil; 54 Functional Imaging Unit, Center for Diagnostic Radiology, Unviersity Medicine Greifswald, Greifswald, Greeifswald Germany; 55 Institute of Physics, University of Campinas, Campinas, Campinas Brazil; 56 Waisman Center, University of Wisconsin, Madison, WI USA; 57 Program in Biomedical Informatics, Stanford University, Stanford, California USA; 58 SRI International, Menlo Park, CA USA; 59 Department of Psychiatry and Behavioral Sciences, Stanford University, Stanford, CA USA; 60 PUCRS, Porto Alegre, Rio Grande do Sul Brazil; 61 Imaging Genetics Center, Mark and Mary Stevens Neuroimagng and Informatics Institute, Keck School of Medicine of University of Southern California, Marina del Rey, CA USA; 62 Département d’informatique, Université de Sherbrooke, Sherbrooke, Québec Canada

## Abstract

I1 Introduction to the 2015 Brainhack Proceedings

R. Cameron Craddock, Pierre Bellec, Daniel S. Margules, B. Nolan Nichols, Jörg P. Pfannmöller

A1 Distributed collaboration: the case for the enhancement of Brainspell’s interface

AmanPreet Badhwar, David Kennedy, Jean-Baptiste Poline, Roberto Toro

A2 Advancing open science through NiData

Ben Cipollini, Ariel Rokem

A3 Integrating the Brain Imaging Data Structure (BIDS) standard into C-PAC

Daniel Clark, Krzysztof J. Gorgolewski, R. Cameron Craddock

A4 Optimized implementations of voxel-wise degree centrality and local functional connectivity density mapping in AFNI

R. Cameron Craddock, Daniel J. Clark

A5 LORIS: DICOM anonymizer

Samir Das, Cécile Madjar, Ayan Sengupta, Zia Mohades

A6 Automatic extraction of academic collaborations in neuroimaging

Sebastien Dery

A7 NiftyView: a zero-footprint web application for viewing DICOM and NIfTI files

Weiran Deng

A8 Human Connectome Project Minimal Preprocessing Pipelines to Nipype

Eric Earl, Damion V. Demeter, Kate Mills, Glad Mihai, Luka Ruzic, Nick Ketz, Andrew Reineberg, Marianne C. Reddan, Anne-Lise Goddings, Javier Gonzalez-Castillo, Krzysztof J. Gorgolewski

A9 Generating music with resting-state fMRI data

Caroline Froehlich, Gil Dekel, Daniel S. Margulies, R. Cameron Craddock

A10 Highly comparable time-series analysis in Nitime

Ben D. Fulcher

A11 Nipype interfaces in CBRAIN

Tristan Glatard, Samir Das, Reza Adalat, Natacha Beck, Rémi Bernard, Najmeh Khalili-Mahani, Pierre Rioux, Marc-Étienne Rousseau, Alan C. Evans

A12 DueCredit: automated collection of citations for software, methods, and data

Yaroslav O. Halchenko, Matteo Visconti di Oleggio Castello

A13 Open source low-cost device to register dog’s heart rate and tail movement

Raúl Hernández-Pérez, Edgar A. Morales, Laura V. Cuaya

A14 Calculating the Laterality Index Using FSL for Stroke Neuroimaging Data

Kaori L. Ito, Sook-Lei Liew

A15 Wrapping FreeSurfer 6 for use in high-performance computing environments

Hans J. Johnson

A16 Facilitating big data meta-analyses for clinical neuroimaging through ENIGMA wrapper scripts

Erik Kan, Julia Anglin, Michael Borich, Neda Jahanshad, Paul Thompson, Sook-Lei Liew

A17 A cortical surface-based geodesic distance package for Python

Daniel S Margulies, Marcel Falkiewicz, Julia M Huntenburg

A18 Sharing data in the cloud

David O’Connor, Daniel J. Clark, Michael P. Milham, R. Cameron Craddock

A19 Detecting task-based fMRI compliance using plan abandonment techniques

Ramon Fraga Pereira, Anibal Sólon Heinsfeld, Alexandre Rosa Franco, Augusto Buchweitz, Felipe Meneguzzi

A20 Self-organization and brain function

Jörg P. Pfannmöller, Rickson Mesquita, Luis C.T. Herrera, Daniela Dentico

A21 The Neuroimaging Data Model (NIDM) API

Vanessa Sochat, B Nolan Nichols

A22 NeuroView: a customizable browser-base utility

Anibal Sólon Heinsfeld, Alexandre Rosa Franco, Augusto Buchweitz, Felipe Meneguzzi

A23 DIPY: Brain tissue classification

Julio E. Villalon-Reina, Eleftherios Garyfallidis

## I1 Introduction to the 2015 Brainhack Proceedings

### R. Cameron Craddock^1,2^, Pierre Bellec^3,4^, Daniel S. Margules^5^, B. Nolan Nichols^6,7^, Jörg P. Pfannmöller^8^

#### ^1^Computational Neuroimaging Lab, Center for Biomedical Imaging and Neuromodulation, Nathan Kline Institute for Psychiatric Research, Orangeburg, NY, USA; ^2^Center for the Developing Brain, Child Mind Institute, New York, NY, USA; ^3^Centre de Recherche, Institut Universitaire de Gériatrie de Montréal, Montréal, Quebec, Canada; ^4^ Department of computer science and operational research, University of Montreal, Montreal, Canada; ^5^Max Planck Research Group for Neuroanatomy & Connectivity, Max Planck Institute for Human Cognitive and Brain Sciences, Leipzig, Germany; ^6^SRI International, Menlo Park, CA, USA; ^7^Department of Psychiatry and Behavioral Sciences, Stanford University, Stanford, CA, USA; ^8^Functional Imaging Unit, Center for Diagnostic Radiology, University Medicine Greifswald, Greifswald, Germany

##### **Correspondence:** R. Cameron Craddock (ccraddock@nki.rfmh.org) – Computational Neuroimaging Lab, Center for Biomedical Imaging and Neuromodulation, Nathan Kline Institute for Psychiatric Research, Orangeburg, NY, USA

Brainhack — a novel conference model for the open neuroscience research community [1] — exploded in 2015. With three-day events in Honolulu (June), Montréal (July), and across the Americas (eight participating sites in October) [http://events.brainhack.org/], a community that first began only a few years ago around the shared spirit of collaboration and an ethos of open science has taken resolute form.

As Brainhack events were founded on the principle that content should emerge through the onsite interaction of participants, the innovative event structure demanded a different publication form. Inverting the model of conference proceedings, where submissions are triaged in preparation for the meeting, we developed the Brainhack Proceedings to rather mark the achievements, outputs, and ideas that emerged as the meeting’s result.

Post-conference papers were solicited from participants at any of the events held in 2015. All submissions were peer-reviewed in the Brainhack Proceedings Github repository [https://github.com/Brainhack-Proceedings-2015] using an innovative open-review process. In keeping with the culture of Brainhack, we took advantage of the open platform provided by Github [http://github.com] to encourage a productive dialogue between authors and reviewers.

This first issue of Brainhack Proceedings includes 23 project papers — presenting an overview of the broad range of interests, content, and achievements that converged at Brainhack events this past year. With at least four international events scheduled for 2016 [http://events.brainhack.org], we hope that this publication format will provide an ongoing record of the growth within our community. Snapshots of all the projects and supporting information can be found in the *GigaScience*, GigaDB, repository [2].

For more information visit the Brainhack home page [http://brainhack.org].

**References**

1. Craddock, R.C, Margulies, D.S., Bellec, P., Nichols, B.N., Alcauter, S., A. Barrios, F., … Xu, T. (2016). Brainhack: a collaborative workshop for the open neuroscience community. *GigaScience*, *5*(1), 16. http://dx.doi.org/10.1186/s13742-016-0121-x

2. Brainhack Proceedings (2016): Dataset collection from the 2015 Brainhack Proceedings GigaScience Database. http://dx.doi.org/10.5524/100215

## A1 Distributed collaboration: the case for the enhancement of Brainspell’s interface

### AmanPreet Badhwar^1,2^, David Kennedy^3^, Jean-Baptiste Poline^4^, Roberto Toro^5^

#### ^1^Centre de Recherche, Institut Universitaire de Gériatrie de Montréal, Montréal, Quebec, Canada; ^2^Université de Montréal, Montréal, Quebec, Canada; ^3^University of Massachusetts Medical School, Worcester, MA, USA; ^4^University of California, Berkeley, CA, USA; ^5^Institut Pasteur, Paris, France

##### **Correspondence:** Roberto Toro (rto@pasteur.fr) – Institut Pasteur, Paris, France

**Introduction**

The past several decades have seen an explosive growth in the number of published neuroimaging studies. In concert, the demand for freely available and openly accessible ‘study data’, that would facilitate future reanalysis, meta-analysis, hypothesis testing and repurposing has also soared. Here we report on developments made to Brainspell[1] one of several web-based initiatives (e.g. BrainMap[2], NeuroVault[3], Neurosynth[4]) that allow individuals to search through and organize massive numbers of neuroimaging studies and results in meaningful ways.

Distinct from other databases, Brainspell [http://brainspell.org] is the first web-based initiative to allow users to manually annotate and curate machine-parsed data, as well as manually extend the database via its crowdsourcing user interface. The goal of our Brainhack project was to improve Brainspell’s interface. We worked to (a) provide supplementary manual data edit options (b) facilitate efficient manual database extension, and (c) aid meaningful organization of data.

**Approach**

We used GitHub to manage the client and server code, and to coordinate its development.

**Results**

**Supplementary manual data edit options**

In the original version of Brainspell, users were able to edit experiment (table) title, caption and coordinates for each article. We added four supplementary options. In particular, users are now provided with enhanced ‘edit feedback’:

- Feedback indicating when a field is editable or has been successfully saved. Editable text fields now turn light grey, while a successfully stored field loses its coloring. Storage of fields can now be triggered by a tab key or by clicking elsewhere, in addition to hitting return.

Users are also provided with additional edit options, specifically, the ability to:

- Add symbols to the title and caption fields.
- Remove empty tables.
- Add and remove rows from a table.

**Database extension**

While users were previously able to add new articles and their coordinate tables, the process was labor- and time-intensive, since each value had to be manually entered. We implemented a more efficient method to edit tables:

- Addition of an *Import* link to each table. When clicked it opens a popup window where comma-separated text can be entered and parsed.

**Meaningful organization of data**

**Fig. 1 (abstract A1).**
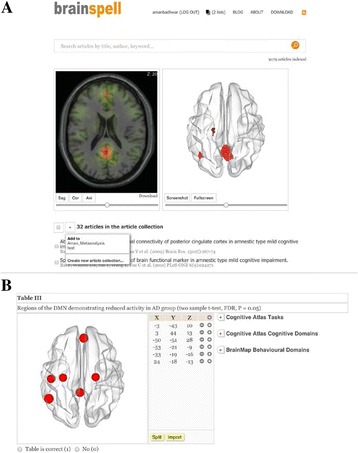
3D volume and mesh showing the aggregated locations of a user/peer-defined collection (Aman_Metaanalysis) containing 32 articles. This user has a total of two collections (or 2 lists), as indicated on the header row. The second collection is named ‘test’. **b** Highlighted in yellow are the Split and Import links associated with each table in Brainspell. Note: With the exception of the Download link, peer-login is required to access all mentioned Brainspell enhancements

Potential shortcomings of neuroimaging databases employing automatic coordinate data extraction is their inability to segregate (i) multiple contrasts (e.g. within group, inter-group), and (ii) significant versus nonsignificant coordinates, when present in a single table. The following options were added to facilitate non-ambiguous data organization (see Fig. 1):

- Addition of a *Split* link to each table.
- Fine-tuning the *Split* link enhancement to allow more than ten splits.
- Option to add articles lacking PMID (or user-specific articles).
- Addition of a *Download* link to each article. When clicked it downloads article title, reference, abstract, and tables.
- Creation of ‘article collection’ functionality. Users can now store the results of their search into article collections. Clicking on an existing collection brings back the corresponding articles and re-computes the 3D volume and mesh of the aggregated locations. Users can create and edit multiple collections.

**Conclusion**

We performed ten enhancements to Brainspell and provided instructions of use in Brainspell’s wiki. We tested these enhancements on Safari, Firefox and Chrome. Moreover, 25 articles were manually added to Brainspell as part of our extended beta testing phase. Our goal with these enhancements was to extend the functionality, and ease of use of Brainspell for curating machine-parsed neuroimaging data from a wide database of studies.

During January 15 to February 5, 2016 alone, Brainspell was used in 282 sessions by 133 users who watched 1421 pages. Moreover, Brainspell was forked to “BIDS-collaborative/Brainspell” which itself was forked by approximately 10 data-science students to extend the platform.

**Availability of supporting data**

More information about this project can be found at: http://github.com/r03ert0/brainspell-brainhack.

**Competing interests**

None.

**Author’s contributions**

RT developed Brainspell. AB, DK, and JBP suggested enhancements and performed beta testing. AB and RT wrote the report.

**Acknowledgements**

Report from 2015 OHBM Hackathon (HI). The authors would like to thank the organizers and attendees of Brainhack 2015 OHBM Hackathon.

**References**

1. Toro R. brainspell. Figshare; 2014. doi:10.6084/m9.figshare.963146.v1.

2. Fox PT, Mikiten S, Davis G, Lancaster JL. BrainMap: A Database of Human Functional Brian Mapping. In: Thatcher RW, Hallett M, Zeffiro T, John ER, Huerta M, editors.Functional Neuroimaging: Technical Foundations. Cambridge, Massachusetts: Academic Press; 1994. p. 95–105.

3. Gorgolewski KJ, Varoquaux G, Rivera G, Schwarz Y, Ghosh SS, Maumet C, Sochat VV, Nichols TE, Poldrack RA, Poline JB, Yarkoni T, Margulies DS. NeuroVault.org: a web-based repository for collecting and sharing unthresholded statistical maps of the human brain. Front Neuroinform. 2015; 9.

4. Yarkoni T, Poldrack RA, Nichols TE, Van Essen DC, Wager TD. Large-scale automated synthesis of human functional neuroimaging data. Nat Methods. 2011; 8: 665–670.

## A2 Advancing open science through NiData

### Ben Cipollini^1^, Ariel Rokem^2^

#### ^1^University of California, San Diego, La Jolla, CA, USA; ^2^University of Washington, Seattle, WA, USA

##### **Correspondence:** Ben Cipollini (bcipolli@ucsd.edu) – University of California, San Diego, La Jolla, CA, USA

**Introduction**

The goal of this project is to improve accessibility of open datasets by curating them. “NiData” aims to provide a common interface for documentation, downloads, and examples to all open neuroimaging datasets, making data usable for experts and non-experts alike.

**Approach**

Open datasets promise to allow more thorough analysis of hard-to-collect data and re-analysis using state- of-the-art analysis methods. However, open datasets are not truly open unless they are easy to find, simple to access, and have sufficient documentation for use. Currently, publicly available data in neuroscience are scattered across a number of websites and databases, without a common data format, no common method for data access, and varying levels of documentation. Datasets are being uploaded to public databases through a number of initiatives, including OpenFMRI [1] and NITRC [2] In addition, there are funded efforts for collecting data explicitly for the purpose of public sharing – most visibly in the Human Connectome Project (HCP) [3] - but also in the Pediatric Imaging, Neurocognition and Genetics (PING) study [4] There are a number of funded efforts to collect old data and re-release as public databases, notably the INDI [5] efforts (which include the popular ABIDE [6] and functional connectomes 1000 datasets [7]). The BRAIN initiative [8] aims to collect data that will be a challenge to store, let alone analyze. There are even online journals focused on publishing datasets (e.g. Nature Scientific Data), or with options to release data (e.g. F1000 “Data Notes”).

NiData is a Python package that provides a single interface accessing data from a variety of open data sources. The software framework makes it easy to add new data sources, simple to define and to provide access to multiple datasets from a single data source. Software dependencies are managed on a per-dataset basis, allowing downloads and examples to use any public packages without requiring installation of packages required by unused datasets. The interface also allows selective download of data (by subject or type) and caches files locally, allowing easy management of big datasets.

**Results**

We focused on exposing new methods for downloading data from the HCP, supporting access via Amazon S3 and HTTP/XNAT [9]. We were able to provide a downloader that accepts login credentials and downloads files locally. We created an example that interacts with DIPY [10] to produce diffusion imaging results on a single subject from the HCP. We also worked at collecting common data sources, as well as individual datasets stored at each data source, into NiData’s “data sources” wiki page. We incorporated downloads, documentation, and examples from the nilearn package and began discussion of making a more extensible object model.

Since the hackathon, we have created such an object model and migrated all code to use it, and a Sphinx- based website is under development. The current object model makes it easier to write general-purpose fetchers (e.g. HTTP, XNAT, Amazon S3) that can be extended to access specific databases (e.g. COINS [11], LORIS [12], ADNI [13]).

**Conclusions**

Projects like NiData improve curated data access and increase the effectivity of big data projects with open source data.

**Availability of supporting data**

More information about this project can be found at: http://github.com/nidata/nidata

**Competing interests**

None.

**Author’s contributions**

BC and AR wrote the software and the report.

**Acknowledgements**

Report from 2015 OHBM Hackathon (HI). The authors would like to thank the organizers and attendees of the 2015 OHBM Hackathon.

**References**

1. Poldrack RA, Barch DM, Mitchell JP, Wager TD, Wagner AD, Devlin JT, Cumba C, Koyejo O, Milham MP. Toward open sharing of task-based fMRI data: the OpenfMRI project. Front Neuroinform. 2013; 7.

2. Buccigrossi R, Ellisman M, Grethe J, Haselgrove C, Kennedy DN, Martone M, Preuss N, Reynolds K, Sullivan M, Turner J, Wagner K. The Neuroimaging Informatics Tools and Resources Clearinghouse (NITRC). AMIA Annu Symp Proc. 2008.

3. Van Essen DC, Smith SM, Barch DM, Behrens TE, Yacoub E, Ugurbil K, Van Essen D, Barch D, Corbetta M, Goate A, Heath A, Larson-Prior L, Marcus D, Petersen S, Prior F, Province M, Raichle M, Schlaggar B, Shimony J, Snyder A, Adeyemo B, Archie K, Babajani-Feremi A, Bloom N, Bryant JE, Burgess G, Cler E, Coalson T, Curtiss S, Danker S, Denness R, Dierker D, Elam J, Evans T, Feldt C, Fenlon K, Footer O, Glasser M, Gordon E, Gu P, Guilday C, Harms M, Hartley T, Harwell J, Hileman M, Hodge M, Hood L, Horton W, House M, Laumann T, Lugo M, Marion S, Miezin F, Nolan D, Nolan T, Power J, Ramaratnam M, Reid E, Schindler J, Schmitz D, Schweiss C, Serati J, Taylor B, Tobias M, Wilson T, Ugurbil K, Garwood M, Harel N, Lenglet C, Yacoub E, Adriany G, Auerbach E, Moeller S, Strupp J, Smith S, Behrens T, Jenkinson M, Johansen-Berg H, Miller K, Woolrich M, Andersson J, Duff E, Hern,ez M, Jbabdi S, Robinson E, Salimi-Khorshidi R, Sotiropoulos S, Romani GL, Della Penna S, Pizzella V, de Pasquale F, Di Pompeo F, Marzetti L, Perruci G, Bucholz R, Roskos T, Kiser T, Luo QJ, Stout J, Oostenveld R, Beckmann C, Schoffelen JM, Fries P, Michalareas G, Sapiro G, Sporns O, Nichols T, Farber G, Bjork J, Blumensath T, Chang A, Chen L, Feinberg D, Kull L, Wig G, Xu JG, Basser P, Bullmore E, Evans A, Gazzaniga M, Glahn D, Hawrylycz M, Hennig J, Parker G, Poldrack R, Salmelin R. The WU-Minn Human Connectome Project: an overview. Neuroimage. 2013; 80: 62–79.

4. Jernigan TL, Brown TT, Hagler DJ, Akshoomoff N, Bartsch H, Newman E, Thompson WK, Bloss CS, Murray SS, Schork N, Kennedy DN, Kuperman JM, McCabe C, Chung Y, Libiger O, Maddox M, Casey BJ, Chang L, Ernst TM, Frazier JA, Gruen JR, Sowell ER, Kenet T, Kaufmann WE, Mostofsky S, Amaral DG, Dale AM. The Pediatric Imaging, Neurocognition, and Genetics (PING) Data Repository. Neuroimage. 2016; 124: 1149–1154.

5. Mennes M, Biswal BB, Castellanos FX, Milham MP. Making data sharing work: the FCP/INDI experience. Neuroimage. 2013; 82: 683–691.

6. Di Martino A, Yan CG, Li Q, Denio E, Castellanos FX, Alaerts K, Anderson JS, Assaf M, Bookheimer SY, Dapretto M, Deen B, Delmonte S, Dinstein I, Ertl-Wagner B, Fair DA, Gallagher L, Kennedy DP, Keown CL, Keysers C, Lainhart JE, Lord C, Luna B, Menon V, Minshew NJ, Monk CS, Mueller S, Muller RA, Nebel MB, Nigg JT, O’Hearn K, Pelphrey KA, Peltier SJ, Rudie JD, Sunaert S, Thioux M, Tyszka JM, Uddin LQ, Verhoeven JS, Wenderoth N, Wiggins JL, Mostofsky SH, Milham MP. The autism brain imaging data exchange: towards a large-scale evaluation of the intrinsic brain architecture in autism. Mol Psychiatry. 2014; 19: 659–667.

7. Biswal BB, Mennes M, Zuo XN, Gohel S, Kelly C, Smith SM, Beckmann CF, Adelstein JS, Buckner RL, Colcombe S, Dogonowski AM, Ernst M, Fair D, Hampson M, Hoptman MJ, Hyde JS, Kiviniemi VJ, Kotter R, Li SJ, Lin CP, Lowe MJ, Mackay C, Madden DJ, Madsen KH, Margulies DS, Mayberg HS, McMahon K, Monk CS, Mostofsky SH, Nagel BJ, Pekar JJ, Peltier SJ, Petersen SE, Riedl V, Rombouts SA, Rypma B, Schlaggar BL, Schmidt S, Seidler RD, Siegle GJ, Sorg C, Teng GJ, Veijola J, Villringer A, Walter M, Wang L, Weng XC, Whitfield-Gabrieli S, Williamson P, Windischberger C, Zang YF, Zhang HY, Castellanos FX, Milham MP. Toward discovery science of human brain function. Proc Natl Acad Sci USA. 2010; 107: 4734–4739.

8. Insel TR, L,is SC, Collins FS. Research priorities. The NIH BRAIN Initiative. Science. 2013; 340: 687–688.

9. Marcus DS, Olsen TR, Ramaratnam M, Buckner RL. The Extensible Neuroimaging Archive Toolkit: an informatics platform for managing, exploring, and sharing neuroimaging data. Neuroinformatics. 2007; 5.

10. Garyfallidis E, Brett M, Amirbekian B, Rokem A, van der Walt S, Descoteaux M, Nimmo-Smith I. Dipy, a library for the analysis of diffusion MRI data. Front Neuroinform. 2014; 8.

11. Scott A, Courtney W, Wood D, de la Garza R, Lane S, King M, Wang R, Roberts J, Turner JA, Calhoun VD. COINS: An Innovative Informatics and Neuroimaging Tool Suite Built for Large Heterogeneous Datasets. Front Neuroinform. 2011; 5.

12. Das S, Zijdenbos AP, Harlap J, Vins D, Evans AC. LORIS: a web-based data management system for multi-center studies. Front Neuroinform. 2011; 5.

13. Jack CR, Bernstein MA, Fox NC, Thompson P, Alex,er G, Harvey D, Borowski B, Britson PJ, L Whitwell J, Ward C, Dale AM, Felmlee JP, Gunter JL, Hill DL, Killiany R, Schuff N, Fox-Bosetti S, Lin C, Studholme C, DeCarli CS, Krueger G, Ward HA, Metzger GJ, Scott KT, Mallozzi R, Blezek D, Levy J, Debbins JP, Fleisher AS, Albert M, Green R, Bartzokis G, Glover G, Mugler J, Weiner MW. The Alzheimer’s Disease Neuroimaging Initiative (ADNI): MRI methods. J Magn Reson Imaging. 2008; 27: 685–691.

## A3 Integrating the Brain Imaging Data Structure (BIDS) standard into C-PAC

### Daniel Clark^1^, Krzysztof J. Gorgolewski^2^, R. Cameron Craddock^1,3^

#### ^1^Center for the Developing Brain, Child Mind Institute, New York, NY, USA; ^2^Poldrack Lab, Department of Psychology, Stanford University, Stanford, CA, USA; ^3^Computational Neuroimaging Lab, Center for Biomedical Imaging and Neuromodulation, Nathan Kline Institute for Psychiatric Research, Orangeburg, NY, USA

##### **Correspondence:** Daniel Clark (daniel.clark@childmind.org) – Center for the Developing Brain, Child Mind Institute, New York, NY, USA

**Introduction**

Data acquired during neuroimaging experiments can be organized in many ways. This stems from differences in scanner software, various DICOM and NIFTI tools, and custom data organizing scripts within different laboratories. The Brain Imaging Data Structure (BIDS) specification [1] provides a simple, straightforward solution to this problem by introducing an intuitive standard for neuroimaging data organization. The widespread adoption of BIDS can be facilitated through incorporating this standard into software projects used for neuroimaging analysis. These software packages will in turn benefit from the homogenous data structure and ease of specifying data acquisition parameters afforded by BIDS. The goal of this Brainhack project was to integrate BIDS into the Configurable Pipeline for the Analysis of Connectomes (C-PAC) [2] a Python Package? built on Nipype [3] for the high-throughput analysis of resting state fMRI data.

**Approach**

Processing data with C-PAC begins with specifying the paths of the anatomical and functional files to be processed, along with their corresponding acquisition parameters. This is accomplished in a semi-automatic procedure in which the user specifies templates that describe the file organization and then a script walks this structure to find the data. The resulting subject list can then be partnered with a pipeline configuration and submitted to C-PAC for processing. We extended this functionality to natively understand BIDS, so that data that conforms to this standard can be configured to run through C-PAC with minimal user intervention.

*C-PAC with BIDS*

A BIDS flag was added to the subject list builder along with a text box for the user to specify the base directory of the data file structure. The BIDS file hierarchy is then traversed to build anatomical and functional file pattern templates. These templates are returned to the main subject list builder function, which runs the same way as if using user specified file path templates. This approach minimized modifications to the data-gathering algorithm while providing for a robust way to ensure all data is found and returned properly. Additional scanning parameters that are required to complete the processing (repetition time, slice timing information, etc.) are read from BIDS specified JSON files that are stored alongside the imaging data.

The new implementation takes advantage of one of many BIDS utilities openly available: the BIDS meta-data tool [4] [https://github.com/INCF/bidsutils] This tool provides the subject, session, and run-level indicators to the builder without needing the user to manually enter any keywords; it takes advantage of the fixed organization scheme and the presence of JSON files to deliver all of this information reliably and efficiently. The tool is written in Python, which provided for easy integration into the C-PAC source code. It works for BIDS datasets stored locally as well as those available through remotely through Amazon S3.

**Results**

**Fig. 2 (abstract A3).**
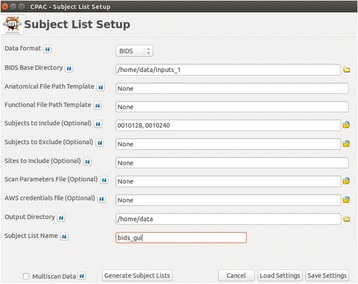
C-PAC subject list builder using BIDS directory

The updated C-PAC GUI reflects the “BIDS” and “Custom” options - as seen in Fig. 2 - with the “Custom” option allowing users to specify their data structure as in previous versions of C-PAC. In the future this option would be more elegantly displayed via a radio button with the input fields dynamically changing to reflect the type of input desired.

The code changes were fairly straightforward and were cleanly inserted into the current builder module [https://github.com/FCP-INDI/C-PAC/blob/test_dev/CPAC/utils/build_sublist.py] The implementation developed during Brainhack is feature full, but will require more testing in the future.

**Conclusions**

Incorporating the BIDS subject list builder into C-PAC is a great step forward in bringing the standard to a broader audience. Throughout the integration process, other technologies were discovered that could further enable input data gathering across a wide range of file system types, including FTP, SFTP, Zip, S3, and an array of virtual filesystems. With further development, the overhead of preprocessing one's own neuroimaging data for discovery science can be minimized so scientists can focus on the results.

**Availability of supporting data**

More information about this project can be found at: https://bids.neuroimaging.io.

**Competing interests**

None.

**Author’s contributions**

RCC and KJG provided supervision and reference, DJC and KJG wrote the software, DJC and KJG performed tests, and DJC wrote the report.

**Acknowledgements**

Report from 2015 Brainhack Americas (MX). The authors would like to thank the organizers and attendees of Brainhack MX and the developers of C-PAC. This project was funded in part by an Educational Research Grant from Amazon Web Services.

**References**

1. Gorgolewski KJ, Poline JB, Keator DB, Nichols BN, Auer T, Craddock RC, Fl,in G, Ghosh SS, Sochat VV, Rokem A, Halchenko YO, Hanke M, Haselgrove C, Helmer K, Maumet C, Nichols TE, Turner JA, Das S, Kennedy DN, Poldrack RA. Brain Imaging Data Structure - a new standard for describing and organizing human neuroimaging data. Frontiers in Neuroscience.

2. Craddock RC, Sikka S, Cheung B, Khanuja R, Ghosh SS, Yan CG, Li Q, Lurie D, Vogelstein J, Burns R, Colcombe S, Mennes M, Kelly C, Di Martino A, Castellanos FX, Milham MP. Towards Automated Analysis of Connectomes: The Configurable Pipeline for the Analysis of Connectomes (C-PAC). Frontiers in Neuroinformatics. 2013.

3. Gorgolewski K, Burns CD, Madison C, Clark D, Halchenko Y), Waskom ML, Ghosh SS. Nipype: a flexible, lightweight and extensible neuroimaging data processing framework in python. Front Neuroinform. 2011; 5.

4. Gorgolewski KJ. bidsutils. GitHub; 2015. https://github.com/INCF/bidsutils.

## A4 Optimized implementations of voxel-wise degree centrality and local functional connectivity density mapping in AFNI

### R. Cameron Craddock^1,2^, Daniel J. Clark^2^

#### ^1^Computational Neuroimaging Lab, Center for Biomedical Imaging and Neuromodulation, Nathan Kline Institute for Psychiatric Research, Orangeburg, NY, USA; ^2^Center for the Developing Brain, Child Mind Institute, New York, NY, USA

##### **Correspondence:** R. Cameron Craddock (ccraddock@nki.rfmh.org) – Computational Neuroimaging Lab, Center for Biomedical Imaging and Neuromodulation, Nathan Kline Institute for Psychiatric Research, Orangeburg, NY, USA

**Introduction**

Degree centrality (DC) [1] and local functional connectivity density (lFCD) [2] are statistics calculated from brain connectivity graphs that measure how important a brain region is to the graph. DC (a.k.a. global functional connectivity density [2]) is calculated as the number of connections a region has with the rest of the brain (binary DC), or the sum of weights for those connections (weighted DC) [1]. lFCD was developed to be a surrogate measure of DC that is faster to calculate by restricting its computation to regions that are spatially adjacent [2]. Although both of these measures are popular for investigating inter-individual variation in brain connectivity, efficient neuroimaging tools for computing them are scarce. The goal of this Brainhack project was to contribute optimized implementations of these algorithms to the widely used, open source, AFNI software package [3]

**Approach**

Tools for calculating DC (3dDegreeCentrality) and lFCD (3dLFCD) were implemented by modifying the C source code of AFNI’s 3dAutoTcorrelate tool. 3dAutoTcorrelate calculates the voxel X voxel correlation matrix for a dataset and includes most of the functionality we require, including support for OpenMP [4] multithreading to improve calculation time, the ability to restrict the calculation using a user-supplied or auto-calculated mask, and support for both Pearson’s and Spearman correlation.

*3dDegreeCentrality*

Calculating DC is straightforward and is quick when a correlation threshold is used. In this scenario, each of the .5*N_*vox*_/(N_vox_-1) unique correlations are calculated, and if they exceed a user specified threshold (default threshold = 0.0) the binary and weighted DC value for each of the voxels involved in the calculation are incremented. The procedure is trickier if sparsity thresholding is used, where the top P% of connections are included in the calculation. This requires that a large number of the connections be retained and ranked - consuming substantial memory and computation. We optimize this procedure with a histogram and adaptive thresholding. If a correlation exceeds threshold it is added to a 50-bin histogram (array of linked lists). If it is determined that the lowest bin of the histogram is not needed to meet the sparsity goal, the threshold is increased by the bin-width and the bin is discarded. Once all of the correlations have been calculated, the histogram is traversed from high to low, incorporating connections into binary and weighted DC until a bin is encountered that would push the number of retained connections over the desired sparsity. This bin’s values are sorted into a 100-bin histogram that is likewise traversed until the sparsity threshold is met or exceeded. The number of bins in the histograms affects the computation time and determines the precision with which ties between voxel values are broken. A greater number of bins allow the sparsity threshold to be determined more precisely but will take longer to converge. Fewer bins will result in faster computation but will increase the tendency of the algorithm to return more voxels than requested. The chosen parameters enable ties to be broken with a precision of 1.0/(50*100), which in our experience offers quick convergence and a good approximation of the desired sparsity.

*3dLFCD*

lFCD was calculating using a region growing algorithm in which face-, side-, and corner-touching voxels are iteratively added to the cluster if their correlation with the target voxel exceeds a threshold (default threshold = 0.0). Although lFCD was originally defined as the number of voxels locally connected to the target, we also included a weighted version.

*Validation*

Outputs from the newly developed tools were benchmarked to Python implementations of these measures from the Configurable Pipeline for the Analysis of Connectomes (C-PAC) [5] using the publicly shared Intrinsic Brain Activity Test-Retest (IBATRT) dataset from the Consortium for Reliability and Reproducibility [6].

**Results**

**Fig. 3 (abstract A4).**
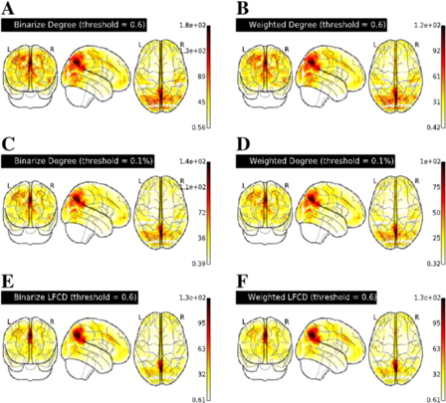
Whole brain maps of binarized and weighted degree centrality calculated with a correlation threshold of 0.6 (**a**-**b**) and sparsity threshold of 0.1 % (**c**-**d**) and binarized and weighted lFCD calculated with a correlation threshold of 0.6 (**e**-**f**) averaged across maps calculated the first resting state scan of the first scanning session for all 36 participants’ data from the IBATRT data

AFNI tools were developed for calculating lFCD and DC from functional neuroimaging data and have been submitted for inclusion into AFNI. LFCD and DC maps from the test dataset (illustrated in Fig. 3) are highly similar to those calculated using C-PAC (spatial concordance correlation [7] 0.99) but required substantially less time and memory (see Table 1).

**Conclusions**

Optimized versions of lFCD and DC achieved 4x to 10x decreases in computation time compared to C-PAC’s Python implementation and decreased the memory footprint to less than 1 gigabyte. These improvements will dramatically increase the size of Connectomes analyses that can be performed using conventional workstations. Making this implementation available through AFNI ensures that it will be available to a wide range of neuroimaging researchers who do not have the wherewithal to implement these algorithms themselves.

**Availability of supporting data**

More information about this project can be found at: http://github.com/ccraddock/afni

**Competing interests**

None.

**Author’s contributions**

RCC and DJC wrote the software, DJC performed tests, and DJC and RCC wrote the report.

**Acknowledgements**

Report from 2015 Brainhack Americas (MX). The authors would like to thank the organizers and attendees of Brainhack MX and the developers of AFNI. This project was funded in part by an Educational Research Grant from Amazon Web Services.

**References**

1. Rubinov M, Sporns O. Complex network measures of brain connectivity: uses and interpretations. Neuroimage. 2010; 52: 1059–1069.

2. Tomasi D, Volkow ND. Functional connectivity density mapping. Proc Natl Acad Sci USA. 2010; 107: 9885–9890.

3. Cox RW. AFNI: software for analysis and visualization of functional magnetic resonance neuroimages. Comput Biomed Res. 1996; 29: 162–173.

4. Dagum Leonardo, Menon Ramesh. OpenMP: an industry standard API for shared-memory programming. Computational Science & Engineering, IEEE. 1998; 5: 46–55.

5. Craddock Cameron, Sikka Sharad, Cheung Brian, Khanuja Ranjeet, Ghosh Satrajit S, Yan Chaogan, Li Qingyang, Lurie Daniel, Vogelstein Joshua, Burns R,al, Colcombe Stanley, Mennes Maarten, Kelly Clare, Di Martino Adriana, Castellanos Francisco Xavier, Milham Michael. Towards Automated Analysis of Connectomes: The Configurable Pipeline for the Analysis of Connectomes (C-PAC). Frontiers in Neuroinformatics. 2013.

6. Xi-Nian Zuo, Jeffrey S Anderson, Pierre Bellec, Rasmus M Birn, Bharat B Biswal, Janusch Blautzik, John CS Breitner, R,y L Buckner, Vince D Calhoun, FXavier Castellanos, Antao Chen, Bing Chen, Jiangtao Chen, Xu Chen, Stanley J Colcombe, William Courtney, RCameron Craddock, Adriana Di Martino, Hao-Ming Dong, Xiaolan Fu, Qiyong Gong, Krzysztof J Gorgolewski, Ying Han, Ye He, Yong He, Erica Ho, Avram Holmes, Xiao-Hui Hou, Jeremy Huckins, Tianzi Jiang, Yi Jiang, William Kelley, Clare Kelly, Margaret King, Stephen M LaConte, Janet E Lainhart, Xu Lei, Hui-Jie Li, Kaiming Li, Kuncheng Li, Qixiang Lin, Dongqiang Liu, Jia Liu, Xun Liu, Yijun Liu, Guangming Lu, Jie Lu, Beatriz Luna, Jing Luo, Daniel Lurie, Ying Mao, Daniel S Margulies, Andrew R Mayer, Thomas Meindl, Mary E Meyer,, Weizhi Nan, Jared A Nielsen, David O’Connor, David Paulsen, Vivek Prabhakaran, Zhigang Qi, Jiang Qiu, Chunhong Shao, Zarrar Shehzad, Weijun Tang, Arno Villringer, Huiling Wang, Kai Wang, Dongtao Wei, Gao-Xia Wei, Xu-Chu Weng, Xuehai Wu, Ting Xu, Ning Yang, Zhi Yang, Yu-Feng Zang, Lei Zhang, Qinglin Zhang, Zhe Zhang, Zhiqiang Zhang, Ke Zhao, Zonglei Zhen, Yuan Zhou, Xing-Ting Zhu, Michael P Milham. An open science resource for establishing reliability and reproducibility in functional connectomics. Scientific Data. 2014; 1: 140049.

7. Lange N, Strother SC, Anderson JR, Nielsen FA, Holmes AP, Kolenda T, Savoy R, Hansen LK. Plurality and resemblance in fMRI data analysis. Neuroimage. 1999; 10: 282–303.

**Table 1 (abstract A4).** Comparison of the time and memory required by the C-PAC and AFNI implementations to calculate DC (sparsity and correlation threshold) and lFCD on the first resting state scan of the first scanning session for all 36 participants’ data in the IBATRT dataset

		r > 0.6		r > 0.6		0.1 % Sparsity	
Impl.	Thr.	Mem GB	TD s	Mem GB	TD s	Mem GB	TD s
Python	1	2.17 (0.078)	67.7 (3.90)	5.62 (0.176)	342.2 (12.25)	2.16 (0.082)	88.3 (6.40)
C	1	0.84 (0.003)	62.6 (9.23)	0.85 (0.002)	86.3 (13.83)	0.86 (0.003)	8.8 (1.27)
C	2	0.86 (0.002)	39.0 (4.62)	0.86 (0.003)	38.2 (0.55)	0.86 (0.003)	5.1 (0.25)
C	4	0.86 (0.003)	18.2 (1.93)	0.87 (0.003)	19.0 (0.45)	0.87 (0.003)	4.3 (0.23)
C	8	0.87 (0.002)	11.2 (0.25)	0.87 (0.000)	11.3 (0.31)	0.87 (0.000)	4.1 (0.15)

Values are averaged across the 36 datasets and presented along with standard deviations in parenthesis. Impl: Implementation, Thr: Number of threads used to process a single dataset, Mem: average (standard deviation) memory in gigabytes used to process a single dataset, T_D_: the average (standard deviation) time in seconds to process a dataset. These statistics were collected on a C3.xlarge Amazon Web Services Elastic Compute Cloud node with 8 hyperthreads and 15 GB of RAM

## A5 LORIS: DICOM anonymizer

### Samir Das^1^, Cécile Madjar^2^, Ayan Sengupta^3^, Zia Mohades^1^

#### ^1^Montréal Neurological Institute, McGill University and Institute of Pscychology, Montréal, Québec, Canada; ^2^Douglas Mental Health Institute, Montréal, Québec, Canada; ^3^Otto-von-Guericke University, Magdeburg, Germany

##### **Correspondence:** Samir Das (samir@acelab.mcgill.edu.ca) – Montréal Neurological Institute, McGill University and Institute of Pscychology, Montréal, Québec, Canada

**Introduction**

The purpose of this Brainhack project was to create a simple application, with the least dependencies, for anonymization of DICOM files directly on a workstation.

Anonymization of DICOM datasets is a requirement before an imaging study can be uploaded in a web-based database system, such as LORIS [1]. Currently, a simple and efficient interface for the anonymization of such imaging datasets, which works on all operating systems and is very light in terms of dependencies, is not available.

**Approach**

**Fig. 4 (abstract A5).**
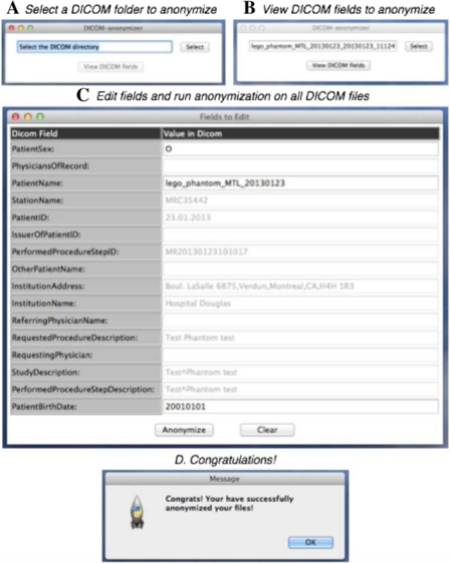
How to use the DICOM anonymizer step by step

Here, we created a DICOM anonymizer that is a simple graphical tool that uses PyDICOM [https://github.com/darcymason/pydicom] package to anonymize DICOM datasets easily on any operating system, with no dependencies except for the default Python and NumPy packages. DICOM anonymizer is available for all UNIX systems (including Mac OS) and can be easily installed on Windows computers as well (see PyDICOM installation [http://pydicom.readthedocs.org/en/latest/getting_started.html]). The GUI (using tkinter [https://wiki.python.org/moin/TkInter]) and the processing pipeline were designed in Python. Executing the anonymizer_gui.py script with a Python compiler will start the program. Figure 4 illustrates how to use the program to anonymize a DICOM study.

**Results**

This graphical tool, designed to be easy-to-use, platform independent and have minimum dependencies, produces two zip files. One zip file includes the original DICOM files and the other contains the anonymized DICOM outputs.

**Conclusions**

The DICOM anonymizer is a simple standalone graphical tool that facilitates anonymization of DICOM datasets on any operating system. These anonymized studies can be uploaded to a web-based database system, such as LORIS, without compromising the patient or participant’s identity.

**Availability of supporting data**

More information about this project can be found at: http://github.com/aces/DICOM_anonymizer.

**Competing interests**

None

**Author’s contributions**

SD, CM, AS, and ZM wrote the software and the report.

**Acknowledgements**

Report from 2015 OHBM Hackathon (HI). The authors would like to thank the organizers and attendees of the 2015 OHBM Hackathon.

**References**

1. Das S. LORIS: a web-based data management system for multi-center studies. FrontNeuroinform. 2011.

## A6 Automatic extraction of academic collaborations in neuroimaging

### Sebastien Dery (sebastien.dery@mail.mcgill.ca)

#### Montreal Neurological Institute, McGill University, Montreal, QC, Canada

**Introduction**

Our ability to quantitatively study large-scale social and behavioural phenomena such as peer influence and confirmation bias within scientific circles rest on quality and relevant data [1] Yet the compilation of specific coauthorship databases are often restricted to certain well-defined fields of study or publication resources, limiting the extent and depth by which investigations can be performed. Ultimately, we aim to understand how the social construct and its underlying dynamics influence the trajectories of scientific endeavors [2] This work is motivated by an interest in observing social patterns, monitoring their evolution, and possibly understanding the emergence and spreading of ideas and their biases in the neuroimaging community; central themes to deciphering facts from opinions. However, before being able to fully investigate and address these fundamental and inherently complex questions, we need to address the extraction and validation of data. The goal of this project was to leverage publicly available information on Google Scholar (GS) to automatically extract coauthorship networks.

**Approach**

The tool can be accessed through a public website [http://cos.dery.xyz]. The site is constructed using a set of openly accessible libraries allowing the display of coauthorship networks as interactive graphs [3] Visitors can peruse a set of pre-computed networks extracted using custom Python scripts designed to crawl GS based on a set of predefined constraints (e.g. search topic, publication journal). The proposed interface offers seamless manipulation to keep interaction straightforward and easy to use. The simplicity of the design aims to reach a maximum number of users, assuming a minimal level of technical knowledge.

*Graph Construction*:

Scholarly citations are commonly found in standardized format, suggesting the structure can be reliably used within an automatic procedure. Moreover, while the result of typical search engines are not structured towards data mining (i.e. mixture of natural language embedded in semi-structured tags and page links), particular combinations of HTML tags and CSS identifiers can be leveraged to extract specific information. This simple scheme allows the reconstruction of large-scale networks of collaborations. Interestingly, Google Scholar also hosts individual pages for authors’ rich with pre-computed metrics of scientific productivity and impact (e.g. cumulative number of citations, h-index, i10-index). This data can be further exploited to structure and highlight part of the network.

*Community Detection*:

Scientific communities were detected using a greedy agglomerative modularity optimization process [4]

*Validation:*

To assess the recovered network’s reliability we performed a spot check on its content. First we examined the accuracy of 100 randomly selected researchers from the network and sought after their departmental affiliation and publication journals to confirm their belonging to the broad field of neuroimaging. The dependence on profile availability injects a strong negative bias. To better appreciate the crawling ability to construct network we further compare with the number of members having a Google Scholar page in the form of a corrected accuracy.

**Results**

**Fig. 5 (abstract A6).**
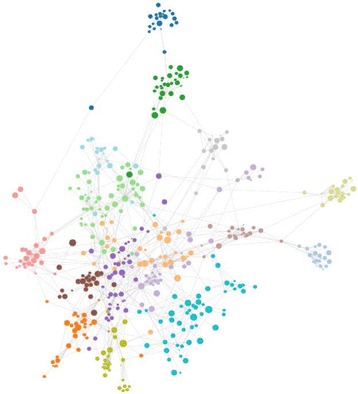
Coauthorship network for the field of neuroimaging. Each disk represent a single researcher with its radius encoding log_10_(Nc), where Nc is the number of citations. Edges stand for a binary relation of coauthorship between two researchers

96 researchers were confirmed to have direct institutional affiliation to neuroscience, psychology, or biomedical engineering departments (see Fig. 5). The remaining 4 randomly selected researchers were found to work in the fields of human genome sequencing, image analysis, nano particles, and pharmacology. Note that these individuals were located on the outskirts of the main graph. To further assess completeness of the network, we compared results with faculty rosters of 5 major neuroimaging institutes (Table 2).

**Conclusions**

Accuracy results suggest a sufficient number of individuals are registered through GS to make it a useful platform of discovery. Meticulous inspection of the grouping suggest that communities typically embed either a geographical or a topical component, that is to say, certain communities are seemingly brought together by either proximity or similarity of interest. With the increasing complexity of science, finding accurate and relevant information on specific topics is a challenging task. We feel that a better appreciation of the wealth and variety of opinions within scientific communities may help enforcing the notion that grand claims require grand evidence.

**Availability of supporting data**

More information about this project can be found at: http://github.com/sderygithub/Clubs-of-Science.

**Competing interests**

None.

**Author’s contributions**

SD wrote the software, performed tests, and wrote the report.

**Acknowledgements**

Report from 2015 Brainhack Montreal. The authors would like to thank the organizers and attendees of Brainhack Montreal.

**References**

1. Freeman LC. The Development of Social Network Analysis: A Study in the Sociology of Science. New York: Empirical Press; 2004.

2. Sarig”ol E, Pfitzner R, Scholtes I, Garas A, Schweitzer F. Predicting scientific success based on coauthorship networks. EPJ Data Science. 2014; 3: 9 + .

3. Holten D, van Wijk JJ. Force-directed Edge Bundling for Graph Visualization. In: Proceedings of the 11th Eurographics/IEEE - VGTC Conference on Visualization, EuroVis’09, Berlin, Germany, 2009. p. 983–998.

4. Blondel VD, Guillaume JL, Lambiotte R, Lefebvre E. Fast unfolding of communities in large networks. Journal of Statistical Mechanics: Theory and Experiment. 2008; 2008: P10008.

**Table 2 (abstract A6).** Completeness study: accuracy between the faculty roster of five major neuroimaging institutes and the neuroimaging network

Institute	Total Count	Recovered	On Google Scholar	Accuracy	Corrected Accuracy
McConnell Brain Imaging Center, Montreal Neurological Institute	12	7	9	58.33 %	77.77 %
Martinos Center for Biomedical Imaging, Harvard University	39	12	22	30.76 %	54.54 %
Cognitive-Neuroimaging Unit, INSERM-CEA, France	15	7	8	46.66 %	87.50 %
Wellcome Trust Center for Neuroimaging, University College London	16	10	11	62.50 %	90.90 %
FMRIB, Oxford University	17	8	11	47.05 %	72.72 %
Totals	99	44	61	49.06 %	76.69 %

## A7 NiftyView: a zero-footprint web application for viewing DICOM and NIfTI files

### Weiran Deng (weirandeng@gmail.com)

#### University of Hawaii John A. Burns School of Medicine, Honolulu, Hawaii, USA, Honolulu, HI, USA

**Introduction**

The purpose of developing yet another web-based image viewer, NiftyView, is to use WebGL to take advantage of the parallel computing power in Graphics Processing Units (GPU) hardware for the acceleration of rendering and processing of medical images in web applications. Although several web-based medical image viewers such as Papaya [https://github.com/rii-mango/Papaya], BrainBrowser [https://brainbrowser.cbrain.mcgill.ca/] and Slice:Drop [http://slicedrop.com] are currently available, the slow performance of the web-based applications is still one of the major limitations of web-based image viewers. NiftyView is a free web application developed in JavaScript. It has zero footprint; only a web browser and an Internet connection are needed to run NiftyView. It’s advantageous over conventional desktop applications in that NiftyView doesn’t require installation and constant updates. The current version supports NIfTI [http://nifti.nimh.nih.gov] and DICOM [http://dicom.nema.org] format. As a minimal image viewer, it’s a convenient tool for users who need a quick and easy tool for viewing medical images. Currently, the beta version of NiftyView is freely available [http://www2.hawaii.edu/~weiran/NiftyView.html].

**Approach**

NiftyView is developed in JavaScript with jQuery [http://jquery.com] for HTML document manipulation and event handling, jQueryUI [http://jqueryui.com] for user interface, and DicomParser [https://github.com/chafey/dicomParser] for parsing DICOM files. It’s compatible with popular web browsers including Internet Explorer, Safari, Firefox, and Opera. Either DICOM or NIfTI files can be loaded by dragging files into the browser window. Loaded images can be displayed in single-slice mode or tiled mode. After loading, images are automatically arranged according to the scan IDs for DICOM files and the file name for NIfTI files, respectively. Current functions include image zooming and adjustment of image brightness and contrast. The number of image columns can be adjusted in the tiled mode to maximize the use of the display space. The contrast and brightness of images can be adjusted by clicking and holding the right mouse button or using a double slider widget in the horizontal tool bar at the top of the window. For proof-of-concept, functions such as pixel windowing and scaling are programmed using WebGL by translating the arithmetic operations in image processing to 3D graphics primitives using WebGL’s programmable shaders. The pixel values of an image are loaded into a frame buffer. A vertex shader is programmed to define vertices corresponding to the coordinates of the image, and a fragment shader is programmed to perform arithmetic operations, which are performed in parallel to a massive number of image pixels.

**Results**

See Figs. 6 and 7.

**Discussion**

One of the major limitations of current web-based image viewers is the slow performance compared to their desktop counterparts.

There are collective efforts in industry to develop new technologies such as WebAssmebly and WebGL to narrow this performance gap. The highly parallel nature in processing image pixels independently allows the use of WebGL to achieve a significant speedup, as shown in this abstract. Currently, there are several similar existing web applications such as Papaya, BrainViewer, and slicedrop.com, which are more mature and offer varieties of features. However, the main goal of the continuing effort in the development of NiftyView is to achieve a high performance for image processing using GPU via WebGL. NiftyView has a minimal boilerplate and can handle a large number of files with relative ease. Future work will be focused on developing a WebGL-accelerated version, adding more image processing features, and adding support of accessing files stored in HIPAA (Health Insurance Portability and Accountability Act) compliant cloud storage services such as Box and Amazon S3. The stable version of NiftyView will be released under a General Public License that allows end users to freely run, modify, and share the program.

**Conclusion**

NiftyView is a free and convenient web application for quick and easy viewing of NIfTI and DICOM medical images. We have shown that a factor of six to eight acceleration can be achieved using WebGL for image processing.

**Availability of supporting data**

More information about this project can be found at: http://www2.hawaii.edu/~weiran/NiftyView.html

**Competing interests**

None.

**Author’s contributions**

WD wrote the code and the report.

**Acknowledgements**

Report from 2015 OHBM Hackathon (HI). We would like to thank the organizers and participants of the 2015 OHBM Hackathon.

**Fig. 6 (abstract A7).**
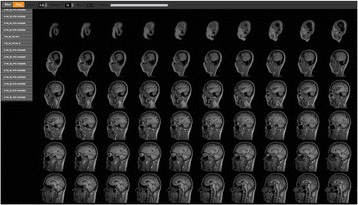
A few sagittal MRI images displayed in titled mode after loading approximately 1,500 DICOM files from 11 MRI scans. It took approximately ten seconds to load all the DICOM files into NiftyView. The images are organized into different vertical tabs by the sequence names stored in the DICOM files

**Fig. 7 (abstract A7).**
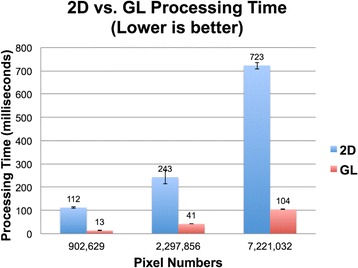
Image of WebGL vs. Canvas Comparison. Shows a comparison of processing time as a function of the number of image pixels in JavaScript (blue) and WebGL (red). WebGL shows a factor of six to eight accelerations

## A8 Human Connectome Project Minimal Preprocessing Pipelines to Nipype

### Eric Earl^1^, Damion V. Demeter^1^, Kate Mills^1^, Glad Mihai^2^, Luka Ruzic^3^, Nick Ketz^4^, Andrew Reineberg^4^, Marianne C. Reddan^4^, Anne-Lise Goddings^5^, Javier Gonzalez-Castillo^6^, Krzysztof J. Gorgolewski^7^

#### ^1^Oregon Health & Science University, Portland, OR, USA; ^2^University of Greifswald, Greifswald, Germany; ^3^Duke Institute for Brain Sciences, Durham, NC,USA; ^4^Department of Psychology and Neuroscience, University of Colorado, Boulder, CO, USA; ^5^Institute of Cognitive Neuroscience, University College London, London, United Kingdom; ^6^Section on Functional Imaging Methods, Laboratory of Brain and Cognition, National Institute of Mental Health, Bethedsa, MD, USA; ^7^Department of Psychology, Stanford University, Stanford, CA, USA

##### **Correspondence:** Eric Earl (earl@ohsu.edu) – Oregon Health & Science University, Portland, OR, USA

**Introduction**

The goal was to convert the Human Connectome Project (HCP) Minimal Preprocessing Pipelines into Nipype code.

The HCP minimal preprocessing pipelines [1] represent a significant advance in image processing pipelines in our time. They provide preprocessed volume and surface data in native and atlas space, for both functional and structural data. Nipype is an open source neuroimaging project for designing imaging pipelines which has been around since 2011 and provides many excellent features for provenance and reliability of processing pipelines [2]. Together, these two pieces of software would allow for a more robust, more flexible synergy of pipeline design and operability.

**Approach**

The first goal was to train the would-be Nipype developers on the Nipype python standards for writing and running interfaces. Once trained, the plan was to implement the HCP scripts into Nipype interfaces from the top-level inward to the sub-level scripts. The secondary goal was to make these sub-level scripts more flexible and require less specific scans to run the pipelines. The collection of nine ultimate pipelines to implement were with or without T1s or T2s and with or without Fieldmap or Reverse-Phase-Encode EPIs as seen in Table 3.

**Results**

Conceptually these goals sounded reasonable enough to do all HCP scripts at once during the hackathon, but the learning and additional setup time was not accounted for, so the scope of the project was too big for two days of on and off coding, even among our eleven developers. Distributing Nipype knowledge from two experts to nine novices over two days was not an easy beginning task, but most of the novices had gained knowledge of Nipype usage by the end of the hackathon. Some work began during the hackathon converting HCP scripts into Nipype pipelines, however not much progress was made due to the unanticipated large scope of work. The second day, an epiphany came about that the original goal, as stated, would have only involved making five top-level wrappers for the five HCP top-level scripts. This also slowed some progress. The secondary goal of generalizing the HCP scripts was discussed, but not thoroughly explored or documented. There has only been some progress in generalization I am aware of in the Neuroimaging Lab (PI: Damien Fair, PA-C, PhD), at OHSU. This turnout of developers during an open hackathon is encouraging and demonstrates the importance of trying to fuse these two systems (Nipype and the HCP scripts) to work together. Work on the repository halted after the hackathon, but the team is still available.

**Conclusions**

More work is needed to truly contribute back to the HCP Pipelines https://github.com/Washington-University/Pipelines. The greatest achievement of the hackathon project was forming a collaborative team of interested Nipype developers who were trained and are ready to continue collaborating across seven institutions. Future work will continue trying to achieve the original goals as stated, but may need an organizer to hold the team accountable to deadlines. To get involved with this project, please contact Eric Earl, earl@ohsu.edu.

**Availability of supporting data**

More information about this project can be found at: https://github.com/ericearl/hcp2nipype-hack2015/

**Competing interests**

None.

**Author’s contributions**

EE wrote the report, EE and all other authors wrote the software.

**Acknowledgements**

Report from 2015 OHBM Hackathon (HI). The authors would like to thank the organizers and attendees of the 2015 OHBM Hackathon.

**References**

1. Glasser MF. The minimal preprocessing pipelines for the Human Connectome Project.. Neuroimage. 2013; 80: 683–691.

2. Gorgolewski K, Burns CD, Madison C, Clark D, Halchenko YO, Waskom ML, Ghosh SS. Nipype: a flexible, lightweight and extensible neuroimaging data processing framework in python. Front Neuroinform. 2011; 5.

**Table 3 (abstract A8).** Nine pipelines to be implemented

EPI	T1	T2	Diffusion Field Map	Reverse Phase Encode EPI
N	N	N	1	0
N	N	0	1	0
N	0	N	1	0
N	N	N	0	N
N	N	0	0	N
N	0	N	0	N
N	N	N	0	0
N	N	0	0	0
N	0	N	0	0

## A9 Generating music with resting-state fMRI data

### Caroline Froehlich^1^, Gil Dekel^3^, Daniel S. Margulies^4^, R. Cameron Craddock^1,2^

#### ^1^Computational Neuroimaging Lab, Center for Biomedical Imaging and Neuromodulation, Nathan Kline Institute for Psychiatric Research, Orangeburg, NY, USA; ^2^Center for the Developing Brain, Child Mind Institute, New York, NY, USA; ^3^City University of New York-Hunter College, New York, NY, USA; ^4^Max Planck Research Group for Neuroanatomy & Connectivity, Max Planck Institute for Human Cognitive and Brain Sciences, Leipzig, Germany

##### **Correspondence:** Caroline Froehlich (cfrohlich@nki.rfmh.org) – Computational Neuroimaging Lab, Center for Biomedical Imaging and Neuromodulation, Nathan Kline Institute for Psychiatric Research, Orangeburg, NY, USA

**Introduction**

Resting-state fMRI (rsfMRI) data generates time courses with unpredictable hills and valleys. People with musical training may notice that, to some degree, it resemble the notes of a musical scale.

Taking advantage of these similarities, and using only rsfMRI data as input, we use basic rules of music theory to transform the data into musical form. Our project is implemented in Python using the midiutil library [https://code.google.com/p/midiutil/].

**Approach**

*Data* We used open rsfMRI from the ABIDE dataset [1] preprocessed by the Preprocessed Connectomes Project [2]. We randomly chose 10 individual datasets preprocessed using C-PAC pipeline [3] with 4 different strategies. To reduce the data dimensionality, we used the CC200 atlas [4] to downsample voxels to 200 regions-of-interest.

**Fig. 8 (abstract A9).**
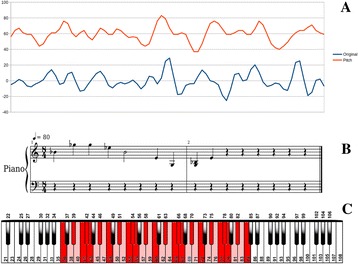
**a** Correspondence between the original time series of one ROI and the generated pitch. **b** The first 10 notes of the same ROI as sheet music. **c** All possible piano keys the brain can play, from 36 to 84 (in pink). We show in red all the possible tones for a C Minor-pentatonic scale, in the range of 36 to 84. In that case, the lowest key is 36. The keys that can be used are: 36, 39, 41, 42, 43, 46, 48, 51, 53, 54, 55, 58, 60, 63, 65, 66, 67, 70, 72, 75, 77, 78, 79, 82, and 84

*Processing:* The 200 fMRI time courses were analyzed to extract pitch, tempo, and volume— 3 important attributes for generating music. For pitch, we mapped the time course amplitudes to Musical Instrument Digital Interface (MIDI) values in the range of 36 to 84, corresponding to piano keys within a pentatonic scale. The key of the scale was determined by the global mean ROI value (calculated across all timepoints and ROIs) using the equation: *(global signal % 49) + 36*. The lowest tone that can be played in a certain key was calculated from *(key % 12) + 36*. The set of tones that could be played were then determined from the lowest tone using a scale. For example, the minor-pentatonic scale’s set of were calculated by adding *0, 3, 5, 7, or 10* to its lowest tone, then skipping to the next octave, and then repeating the process until the value 84 was reached. An fMRI time course was mapped to these possible tones by scaling its amplitude to the range between the smallest and largest tones in the set. If a time point mapped to a tone that was not in the set, it was shifted to the closest allowable tone. An example of allowed set of tones is shown in Fig. 8.

For tempo, we used first temporal derivative for calculating the length of notes, assuming we have 4 lengths (whole, half, quarter and eighth note). In the time course, if the modulus distance between time point *t* and *t + 1* was large, we interpreted it as a fast note (eighth). However, if the distance between *t* and *t + 1* was close to zero, we assumed it is a slow note (whole). Using this approach, we mapped all other notes in between.

We used a naive approach for calculating volume in a way that tackles a problem we had with fast notes: their sound is cut off due to their short duration. A simple way to solve this is to decrease the volume of fast notes. Thus, the faster the note, the lower the volume. While a whole note has volume 100 ([0,100]), an eighth note has volume 50.

Finally, we selected the brain regions that will play. Users complain when two similar brain regions play together. Apparently, the brain produces the same music twice. However, when the regions are distinct, the music is more pleasant. Thus, we used FastICA [5] for choosing brain regions with maximally uncorrelated time courses.

**Results**

A framework for generating music from fMRI data, based on music theory, was developed and implemented as a Python tool yielding several audio files. When listening to the results, we noticed that music differed across individual datasets. However, music generated by the same individual (4 preprocessing strategies) remained similar. Our results sound different from music obtained in a similar study using EEG and fMRI data [6]

**Conclusions**

In this experiment, we established a way of generating music with open fMRI data following some basic music theory principles. This resulted in a somewhat naïve but pleasant musical experience. Our results also demonstrate an interesting possibility for providing feedback from fMRI activity for neurofeedback experiments.

**Availability of Supporting Data**

More information about this project can be found at: https://github.com/carolFrohlich/brain-orchestra

**Competing interests**

None.

**Author’s contributions**

CF wrote the software. GD designed the functions for transforming the data to midi. DSM pick the algorithm that chooses ROIs, and CF and RCC wrote the report.

**Acknowledgements**

Report from 2015 Brainhack Americas (MX). The authors would like to thank the organizers and attendees of Brainhack MX.

**References**

1. Di Martino A, Yan CG, Li Q, Denio E, Castellanos FX, Alaerts K, Anderson JS, Assaf M, Bookheimer SY, Dapretto M, others. The autism brain imaging data exchange: towards a large-scale evaluation of the intrinsic brain architecture in autism. Molecular psychiatry. 2014; 19: 659–667.

2. Craddock RC, Benhajali Y, Chu C, Chouinard F, Evans A, Jakab A, Khundrakpam BS, Lewis JD, Li Q, Milham MP, Yan CG, Bellec P. The Neuro Bureau Preprocessing Initiative: open sharing of preprocessed neuroimaging data and derivatives.Frontiers in Neuroinformatics.

3. Craddock RC, Sikka S, Cheung B, Khanuja R, Ghosh SS, Yan CG, Li Q, Lurie D, Vogelstein J, Burns R, Colcombe SJ, Mennes M, Kelly C, Di Martino A, Castellanos FX, Milham M. Towards Automated Analysis of Connectomes: The Configurable Pipeline for the Analysis of Connectomes (C-PAC). Frontiers in Neuroinformatics. 2013.

4. Craddock RC, James GA, Holtzheimer PE, Hu XP, Mayberg HS. A whole brain fMRI atlas generated via spatially constrained spectral clustering. Human Brain Mapping. 2012; 33: 1914–1928.

5. Pedregosa F, Varoquaux G, Gramfort A, Michel V, Thirion B, Grisel O, Blondel M, Prettenhofer P, Weiss R, Dubourg V, V,erplas J, Passos A, Cournapeau D, Brucher M, Perrot M, Duchesnay E. Scikit-learn: Machine Learning in Python. Journal of Machine Learning Research. 2011; 12: 2825–2830.

6. Lu J, Wu D, Yang H, Luo C, Li C, Yao D. Scale-Free Brain-Wave Music from Simultaneously EEG and fMRI Recordings. PLoS ONE. 2012; 7: 1–11.

## A10 Highly comparable time-series analysis in Nitime

### Ben D. Fulcher (ben.fulcher@monash.edu)

#### Monash Institute of Cognitive and Clinical Neurosciences, Monash University, Melbourne, Australia

**Introduction**

The aim of this project was to demonstrate that an existing Matlab-based package for implementing thousands of time-series analysis methods, hctsa [https://github.com/benfulcher/hctsa] could be extended to a Python-based implementation, for potential future inclusion into Nitime [http://nipy.org/nitime/].

Recent work has contributed a comprehensive library of over 35,000 pieces of diverse time-series data, and over 7,000 unique structural features extracted from hundreds of different time-series analysis methods [1] which can be explored through an associated website [www.comp-engine.org/timeseries] and implemented using the Matlab-based code package, hctsa [https://github.com/benfulcher/hctsa].

The *hctsa* software provides a systematic, algorithmic platform for computing a wide range of structural properties from a single time series, including basic statistics of the distribution, linear correlation structure, stationarity, information theoretic and entropy measures, methods from the physical nonlinear time-series analysis literature, linear and nonlinear model fits, and others. Thus, hctsa can be used to map a time series to a comprehensive vector of interpretable structural features and these features can then be systematically compared to determine and understand the most useful features for a given scientific objective (e.g., features of an EEG signal that help classify different patient groups).

In order to apply highly comparative time-series analysis in the neuroscience community, it would be desirable to implement some time-series analysis methods into Nitime [http://nipy.org/nitime/], a Python-based software package for performing time-series analysis on neuroscience data.

Implementation of useful time-series features into python, and potential integration with Nitime, would not only facilitate their use by the neuroscience community, but also their maintenance and development within an open source framework.

**Approach**

**Fig. 9 (abstract A10).**
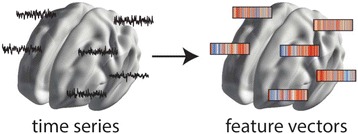
Illustration of the highly comparative approach to time-series data from neuroscience

An illustration of the approach is shown in Fig. 9

Each time series is converted to a vector of thousands of informative features using the *hctsa* package; machine-learning methods can then be used to determine the most useful features (e.g., that best discriminate patient groups, and where in the brain the best discrimination occurs).

In this project, we wanted to demonstrate a feasible pathway for incorporating these useful features into the Nitime package.

**Results**

I successfully implemented a handful of basic time-series analysis functions from Matlab into python using partials (a python function that freezes a given set of input arguments to a more general function).

The proof-of-principle implementation has full support for vectors of data stored in numpy arrays, and basic support for the Nitime data format (extracting the data vector from the Nitime TimeSeries class for evenly sampled data).

**Conclusions**

Our results demonstrate that time-series analysis methods, discovered using the hctsa package [https://github.com/benfulcher/hctsa], can be implemented natively in python in a systematic way, with basic support for the time-series format used in Nitime.

This will help facilitate future work on time-series analysis to be incorporated straightforwardly into this open source environment.

Although there are no plans to reimplement the full *hctsa* feature library in python, our hope is that published work describing useful time-series features (discovered using the *hctsa* library) can also contribute to a Python implementation, to promote its use by the neuroscience community.

**Availability of supporting data**

More information about this project can be found at: https://github.com/benfulcher/hctsa_python

**Competing interests**

None.

**Author’s contributions**

BF wrote the software and the report.

**Acknowledgements**

Report from 2015 OHBM Hackathon (HI). The authors would like to thank the organizers and attendees of the 2015 OHBM Hackathon.

**References**

1. Fulcher Ben D, Little Max A, Jones Nick S. Highly comparative time-series analysis: the empirical structure of time series and their methods. J Roy Soc Interface. 2013; 10: 20130048.

## A11 Nipype interfaces in CBRAIN

### Tristan Glatard^1,2^, Samir Das^1^, Reza Adalat^1^, Natacha Beck^1^, Rémi Bernard^1^, Najmeh Khalili-Mahani^1^, Pierre Rioux^1^, Marc-Étienne Rousseau^1^, Alan C. Evans^1^

#### ^1^McGill Centre for Integrative Neuroscience (MCIN), Ludmer Centre for Neuroinformatics and Mental Health, Montreal Neurological Institute (MNI), McGill University, Montréal, Québec, Canada; ^2^University of Lyon, CNRS, INSERM, CREATIS., Villeurbanne, France

##### **Correspondence:** Tristan Glatard (tristan.glatard@mcgill.ca) – McGill Centre for Integrative Neuroscience (MCIN), Ludmer Centre for Neuroinformatics and Mental Health, Montreal Neurological Institute (MNI), McGill University, Montréal, Québec, Canada

**Introduction**

We aim at the large-scale, automatic sharing of software tools between neuroimaging processing platforms, which will increase the relevance of such platforms by providing them with richer repositories of higher-quality tools. Currently, efforts are hampered by the repetitive porting of the same few tools in different platforms. During the HBM 2015 Hackathon, we worked on the export of software tools from the Nipype workflow engine [1] to the CBRAIN web platform for distributed computing [2]. Nipype includes a large number of tools that would be useful to CBRAIN users.

**Approach**

**Fig. 10 (abstract A11).**
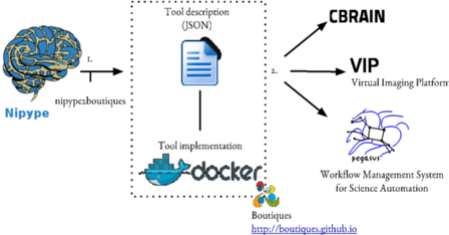
System architecture

We developed nipype2boutiques, a tool to export Nipype interfaces to the “Boutiques” tool description format (step 1. on Fig. 10.). Boutiques descriptions are importable by CBRAIN and other platforms (Virtual Imaging Platform [3] and the Pegasus workflow engine [4]). They point to a Docker image containing the implementation of the tool. nipype2boutiques relies on nipype_cmd a tool to run Nipype Interfaces as Linux command lines. nipype2boutiques parses the inputs and outputs of a Nipype interface and extracts their name, type, description and position on the nipype_cmd command line. nipype2boutiques then generates a Boutiques descriptor pointing to a Docker image where the Nipype interface is available. Once a Nipype interface is exported using nipype2boutiques it can be imported to CBRAIN.

**Results**

We tested nipype2boutiques on a few Nipype interfaces from the FSL Nipype module. We exported 64 FSL tools automatically from Nipype to CBRAIN, and made them available [https://github.com/glatard/boutiques-nipype-fsl]. Limitations remain on the type of Nipype interface that can be exported by nipype2boutiques: in particular, InputMultiPath is? currently not supported, and output files have to be written in the execution directory of the Nipype Interface.

**Conclusions**

We prototyped a software tool to export Nipype Interfaces as Boutiques descriptors, which can be imported by CBRAIN and other platforms. Although the solution is still limited to simple interfaces, we believe that it has the potential to enable fully automatic tool sharing between Nipype and CBRAIN. Future extensions of nipype2boutiques will be published in the Nipype Github repository [https://github.com/nipy/nipype]. We also plan on a tighter integration of Nipype workflows in CBRAIN, following the model adopted in [5].

**Availability of Supporting Data**

More information about this project can be found at: http://cbrain.mcgill.ca.

**Competing interests**

None.

**Author’s contributions**

TG wrote the software and the report; SD contributed to the concept elaboration at the OHBM event, RA, NB, PR and MER provided support on the CBRAIN framework, RB implemented Boutiques in CBRAIN, NKM provided background information on fMRI packages, ACE spearheaded the project.

**Acknowledgements**

Report from 2015 OHBM Hackathon (HI). The authors would like to thank the organizers and attendees of the 2015 OHBM Hackathon.

**References**

1. Gorgolewski Krzysztof, Burns Christopher D, Madison Cindee, Clark Dav, Halchenko Yaroslav O, Waskom Michael L, Ghosh Satrajit S. Nipype: a flexible, lightweight and extensible neuroimaging data processing framework in Python. Frontiers in Neuroinformatics. 2011; 5.

2. Sherif Tarek, Rioux Pierre, Rousseau Marc-Etienne, Kassis Nicolas, Beck Natacha, Adalat Reza, Das Samir, Glatard Tristan, Evans Alan C. CBRAIN: a web-based, distributed computing platform for collaborative neuroimaging research. Frontiers in neuroinformatics. 2014; 8.

3. Glatard T, Lartizien C, BGibaud, Ferreira da Silva R, Forestier G, Cervenansky F, Aless,rini M, Benoit-Cattin H, Bernard O, Camarasu-Pop S, Cerezo N, Clarysse P, Gaignard A, Hugonnard P, Liebgott H, Marache S, Marion A, Montagnat J, Tabary J, Friboulet D. A Virtual Imaging Platform for multi-modality medical image simulation. IEEE Transactions on Medical Imaging. 2013; 32: 110–118.

4. Deelman E, Vahi K, Rynge M, Juve G, Mayani R, da Silva RF. Pegasus in the Cloud: Science Automation through Workflow Technologies. Internet Computing, IEEE. 2016; 20: 70–76.

5. Glatard T, Quirion PO, Adalat R, Beck N, Bernard R, Caron BL, Nguyen Q, Rioux P, Rousseau M-E, Evans AC, Bellec P. Integration between PSOM and CBRAIN for distributed execution of neuroimaging pipelines. In: Meeting of the Organization for Human Brain Mapping, Geneva, Switzerland, OHBM 2016, Geneva, 2016.

## A12 DueCredit: automated collection of citations for software, methods, and data

### Yaroslav O. Halchenko, Matteo Visconti di Oleggio Castello

#### Department of Pscyhological & Brain Sciences, Dartmouth College, Hanover, NH, USA

##### **Correspondence:** Yaroslav O. Halchenko (yoh@onerussian.com) – Department of Pscyhological & Brain Sciences, Dartmouth College, Hanover, NH, USA

**Introduction**

Data analysis software and canonical datasets are the driving force behind many fields of empirical sciences. Despite being of paramount importance, those resources are most often not adequately cited. Although some can consider this a “social” problem, its roots are technical: Users of those resources often are simply not aware of the underlying computational libraries and methods they have been using in their research projects. This in-turn fosters inefficient practices that encourage the development of new projects, instead of contributing to existing established ones. Some projects (e.g. FSL [1]) facilitate citation of the utilized methods, but such efforts are not uniform, and the output is rarely in commonly used citation formats (e.g. BibTeX). DueCredit is a simple framework to embed information about publications or other references within the original code or dataset descriptors. References are automatically reported to the user whenever a given functionality or dataset is being used.

**Approach**

DueCredit is currently available for Python, but we envision extending support to other frameworks (e.g., Matlab, R). Until DueCredit gets adopted natively by the projects, it provides the functionality to “inject” references for 3rd party modules.

For the developer, DueCredit implements a decorator *@due.dcite* that allows to link a method or class to a set of references that can be specified through a doi or BibTeX entry. For example (from PyMVPA):
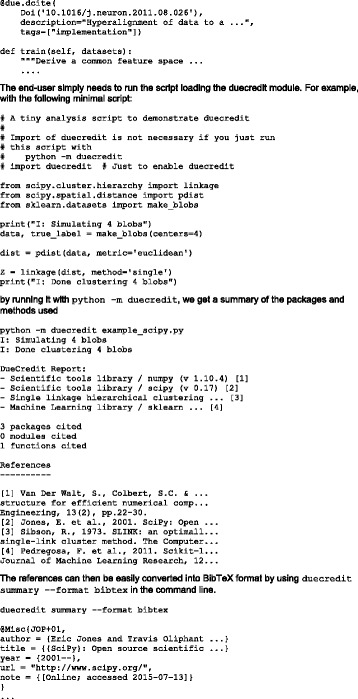


**Results**

The initial release of DueCredit (0.1.0) was implemented during the OHBM 2015 hackathon and uploaded to pypi and is freely available. DueCredit provides a concise API to associate a publication reference with any given module or function. For example: To provide a reference for an entire module the cite function can be used, while functions and methods can be conveniently decorated using dcite. DueCredit comes with a simple demo code, which demonstrates its utility. Running a sample analysis produces a summary of references. At each run, the information is stored in a pickled file, and incremental runs update that file. Thus, DueCredit summary can be used to show that information again or export it as a BibTeX file ready for reuse.

**Conclusions**

DueCredit is in its early stages of development, but two days of team development at the OHBM hackathon were sufficient to establish a usable prototype implementation. Since then, the code-base was further improved and multiple beta-releases followed, expanding the coverage of citable resources (e.g., within scipy, sklearn modules via injections and PyMVPA natively).

**Availability of supporting data**

More information about this project can be found at: https://github.com/duecredit/duecredit

**Competing interests**

None.

**Author’s contributions**

YOH and MVdOC performed the project and wrote the report.

**Acknowledgements**

Report from 2015 OHBM Hackathon (HI). The authors would like to thank the organizers and attendees of the 2015 OHBM Hackathon. This project is supported in part by a grant from the NSF (award 1429999). MVdOC was supported by a Dartmouth Graduate Studies Travel Grant.

**References**

1. Smith Stephen M, Jenkinson Mark, Woolrich Mark W, Beckmann Christian F, Behrens Timothy EJ, Johansen-Berg Heidi, Bannister Peter R, De Luca Marilena, Drobnjak Ivana, Flitney David E, others. Advances in functional and structural MR image analysis and implementation as FSL. Neuroimage. 2004; 23: S208–S219.

## A13 Open source low-cost device to register dog’s heart rate and tail movement

### Raúl Hernández-Pérez, Edgar A. Morales, Laura V. Cuaya

#### Instituto de Neurobiología, Queretaro, Queretaro, Mexico

##### **Correspondence:** Laura V. Cuaya (lauveri.rozen@gmail.com) – Instituto de Neurobiología, Queretaro, Queretaro, Mexico

**Introduction**

In dogs, the perception of an important stimulus can be related to physiological changes such as the heart rate (e.g., in socioemotional situations with humans [1] or dogs [2]) and the movement of their tail (for example, tail-wagging has a bias that depends on the nature of the stimulus, a bias to the left is related to a withdrawal tendency and a bias to the right is related to an approach tendency [3]).

Although heart rate and the tail movement are important gateways to understanding dog cognition, just a few studies report these variables. Perhaps this is related to the difficulty of obtaining records of these variables in natural environments (e.g., parks), the elevated cost of commercial data acquisition hardware (around 5,000 USD [4]) or by nonexistence of a tail-movement registering device. For these reasons, the goal of this Brainhack project is to design and build a low cost device able to register the heart rate and changes in the tail movement in dogs, both in laboratory and in free movement conditions.

**Approach**

We decided to base our design in Arduino hardware for its accessibility and broad use. The materials are detailed in the Table 4.

We designed and 3d printed a PLA case to contain the circuit. The case has a slot to add a strap to fix the device on the dogs back. The program for the Arduino and the model for the case can be downloaded from the GitHub (scripts directory) repository of the project.

In order to assess if the device could reliably get readings from a dog, we tested it in three phases: baseline, stimulation/no-stimulation and free movement. All phases lasted two minutes and were repeated twice on two dogs. In both, baseline and stimulation/no-stimulation, the dog stayed in sphinx position without movement restrictions but under the command “stay”. The stimulation/no-stimulation phase consisted in three interleaved repetitions of two types of conditions, stimulation and no-stimulation, each repetition lasted 20 s. In the stimulation condition the dog owner showed the dog a treat and mentioned the dog’s name. In the free movement condition, the dog walked down a street without any specific command.

## Results

**Fig. 11 (abstract A13).**
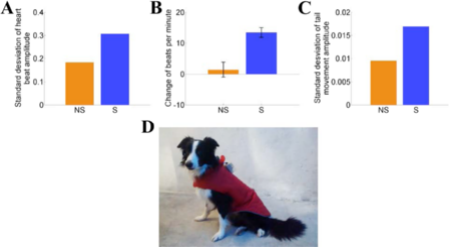
The results shown were obtained from two dogs under two consecutive conditions. Stimulation and No-stimulation. In panels **a**, **b** and **c**, the colors represent the conditions. The panel **a** represents the standard deviation from the mean of the heart beat amplitude. The panel **b** represents the change on the beats per minute on both conditions minus a baseline registered directly from each dog. The vertical lines represent the standard error. The panel **c** represents the standard deviation from the mean of the tail movement. The panel **d** shows one of the registered dogs wearing the device

In the stimulation/no-stimulation phase a Wilcoxon Signed Rank Test revealed statistically significant differences (p < 0.05) between the beats per minute, beat amplitude and the tail movement amplitude (Fig. 11).

By matching the data collected with observations of the movement of the tail, we notice that the data reflects the position of the tail but its resolution depended on the position of the electrode. The data acquired from the free movement condition was affected by the movement and did not seem reliable for testing.

**Conclusions**

We were able to build and test a non-invasive low cost device with the capacity to register the heart rate and the tail movement of dogs. We consider that the addition of a movement sensor could provide additional data to reduce the change on the signal due to movement.

This device can be integrated in future research on dog cognition. It can also be used in shelters and homes to easily measure the responses that dogs present to different sets of stimuli; for example, when a dog is left alone in its house and shows stress (i.e. increased heart rate, preferential tail movement to the left) the dog’s care giver could make changes in the environment to increase the well-being of the dog.

The low cost and the easy access to the materials needed to build the device make it a feasible option to study dog cognition. The results showed that the device could be used to distinguish between two different stimulation conditions.

**Availability of supporting data**

More information about this project can be found at: https://github.com/nekrum/DogVest.

**Competing interests**

None

**Author’s contributions**

LVC generated the idea for the project, made the research, help writing the report and acquire the data. EAM and RH designed the device, build it, wrote the code and helped write the report.

**Acknowledgements**

Report from 2015 Brainhack Americas (MX). We would like to thank the organizers and attendees of Brainhack MX and to the Instituto de Neurobíologia. Especially to Fernando Barrios Alvarez for the invitation and the support on the realization of the project. Laura V. Cuaya, Raul Hern’andez and Edgar Morales are doctoral students from Programa de Doctorado en Ciencias Biom’edicas, Universidad Nacional Aut’onoma de M’exico (UNAM) and received fellowship 407590, 409258 and 215702 from CONACYT.

**References**

1. Palestrini C, Previde EP, Spiezio C, Verga M. Heart rate and behavioural responses of dogs in the Ainsworth’s Strange Situation: A pilot study. Applied Animal Behaviour Science. 2005; 94: 75–88.

2. Siniscalchi M, Lusito R, Vallortigara G, Quaranta A. Seeing Left- or Right-Asymmetric Tail Wagging Produces Different Emotional Responses in Dogs. Current Biology. 2013; 23: 2279–2282.

3. Quaranta A, Siniscalchi M, Vallortigara G. Asymmetric tail-wagging responses by dogs to different emotive stimuli. Current Biology. 2007; 17: R199–R201.

4. PARAGON MEDICAL SUPPLY. Vmed Bluetooth Wireless Veterinary Monitors. 2014.

**Table 4 (abstract A13).** Materials and cost

Materials	Aproximate cost (in USD)
Arduino UNO rev3	20.00
EKG-EMG-shield from Olimex with electrodes	48.00
Vibration sensor from phidgets	11.00
9v rechargeable battery	7.00
SD Card Reader module ARM MCU	1.20
Total	87.20

The table shows most of the materials used and their approximated cost with a local provider. Other materials were used but their cost is negligible

## A14 Calculating the Laterality Index Using FSL for Stroke Neuroimaging Data

### Kaori L. Ito, Sook-Lei Liew

#### Neural Plasticity and Neurorehabilitation Laboratory, Chan Division of Occupational Science and Occupational Therapy, Division of Biokinesiology and Physical Therapy, Keck School of Medicine Department of Neurology, University of Southern California, Los Angeles, CA, USA

##### **Correspondence:** Kaori L. Ito (kaoriito@usc.edu) – Neural Plasticity and Neurorehabilitation Laboratory, Chan Division of Occupational Science and Occupational Therapy, Division of Biokinesiology and Physical Therapy, Keck School of Medicine Department of Neurology, University of Southern California, Los Angeles, CA, USA

**Introduction**

The laterality index (LI) is one way to assess hemispheric dominance in a variety of tasks, such as language, cognitive functions, and changes in laterality in clinical populations, such as after stroke. In stroke neuroimaging, however, an optimal method of calculating the LI remains controversial, largely due to lesion variability in post-stroke brains.

Two main methods of calculating LI have evolved in neuroimaging literature [1] The first, more traditional approach counts the number of active voxels in a given region of interest (ROI) for each hemisphere. This method has been criticized for its inability to account for differences in signal intensity. Hence, a second approach calculates laterality based on the percent signal change within a given region; however, this method also has problems, such as difficulty handling negative values.

A laterality toolbox that addresses some of these issues has been implemented in the statistical neuroimaging analysis package SPM, which provides users with options of using either method, along with more advanced statistical tests for robust LI calculations [2] No such toolbox is yet available for FSL. Therefore, we developed a series of scripts to calculate LI in FSL using both voxel count and percent signal change methods. However, in the interest of space, here we present only results from the more robust method of the two (voxel count method).

**Approach**

We used fMRI data from two groups of stroke participants who either had right or left hemisphere lesions. Participants observed videos of right or left hand actions, and resulting statistical maps were calculated for each individual. The LI was then calculated per participant, based on the number of active voxels within a given anatomically-defined ROI (the inferior frontal gyrus, pars opercularis). Using the *cluster* tool in FSL, we set a threshold on the second-level whole-brain map. We set a range of z-values (z = 1.0, z = 1.5, z = 2.3) to test the effects of different thresholds. We then utilized *fslstats* to determine the total number of active voxels in both left and right hemisphere ROIs. Finally, we calculated LI based on the equation:

$$ \mathrm{L}\mathrm{I} = \left(\mathrm{L}\hbox{-} \mathrm{R}\right)/\left(\mathrm{L} + \mathrm{R}\right) $$

where *L* represents the number of active voxels in the left-hemisphere ROI and *R* is the number of active voxels in the right-hemisphere ROI. This yields a value for LI such that −1 < *LI* < +1, where a positive value indicates left-hemisphere dominance and a negative value indicates right-hemisphere dominance.

**Results/Discussion**

**Fig. 12 (abstract A14).**
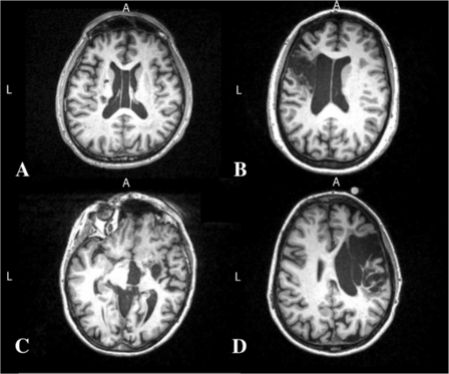
A Comparison Across Different Stroke Lesion Profiles at Maximum Lesion. MRI scans of individuals who sustained **a** subcortical left-hemisphere stroke, **b** cortical left-hemisphere stroke, **c** subcortical right-hemisphere stroke, **d** cortical right-hemisphere stroke

We examined the variability in LI at different z-value thresholds to look at laterality differences in individuals with cortical versus subcortical stroke as well as the affected hemisphere (R vs. L). The LI values of four representative individuals (see Fig. 12) with the following types of stroke were as follows (see Table 5): subcortical left-hemisphere stroke (mean LI = −0.23; right lateralized), subcortical right-hemisphere stroke (mean LI = 0.79; left lateralized), cortical left-hemisphere stroke (mean LI = 0.96, left lateralized), and cortical right-hemisphere stroke (mean LI = 0.94, left lateralized). These LI results corresponded with our whole brain observations (not included here).

Importantly, we notice that the voxel count method is highly dependent on the threshold value: as the threshold increases in stringency, the value of the LI increases. With individuals after stroke, higher thresholds may yield 0 active voxels, leading to a potentially skewed LI (LI = 1).

**Conclusions**

We suggest that stroke neuroimaging might benefit from calculating an average LI across different thresholds (including more lenient thresholds such as z = 1.0), in order to provide a more robust outcome that takes into account threshold dependency. This is especially true for individuals with cortical strokes, where the ROI may overlap with the lesion and yield 0 active voxels. This issue of thresholding, specifically for stroke research, is an interesting question that remains to be addressed further. Our scripts for these calculations may be found online at the NPNL resource page [http://chan.usc.edu/npnl/resources].

**Availability of supporting data**

More information about this project can be found at: http://github.com/npnl/LI_FSL

**Competing interests**

None.

**Author’s contributions**

KLI and SLL wrote the software, performed tests, and wrote the report.

**Acknowledgements**

Report from 2015 Brainhack Americas (LA). The authors would like to thank the organizers and attendees of Brainhack LA and the developers of FSL. This project was funded in part by an Educational Research Grant from Amazon Web Services.

**References**

1. Jansen A, Menke R, Sommer J, Förster AF, Bruchmann S, Hempleman J, Weber B, Knecht S. The assessment of hemispheric lateralization in functional MRI014Robustness and reproducibility. NeuroImage. 2006; 33: 204–217.

2. Wilke M, Lidzba K. LI-tool: A new toolbox to assess lateralization in functional MR-data. Journal of Neuroscience Methods. 2007; 163: 128–136.

**Table 5 (abstract A14).** Laterality index using a voxel-count-based method in FSL: a comparison across different stroke lesion profiles and different thresholds

		Subcortical Lesion			Cortical Lesion		
Side of Stroke Lesion	Z-Score	LH	RH	LI	LH	RH	LI
Left	1	272	284	−0.022	382	22	0.891
	1.5	167	217	−0.130	101	0	1
	2.3	37	123	−0.538	1	0	1
Mean				−0.230			0.964
Right	1	335	68	0.662	509	49	0.824
	1.5	193	29	0.739	318	3	0.981
	2.3	76	1	0.974	216	0	1
Mean				0.792			0.935

## A15 Wrapping FreeSurfer 6 for use in High-performance Computing Environments

### David G. Ellis^1^, Regina E.Y. Kim^2^, Ipek Oguz^3^, Hans J. Johnson^2,3,4^

#### ^1^Department of Biomedical Engineering, The University of Iowa, Iowa City, Iowa, USA; ^2^Department of Pyschiatry, Carver College of Medicine, The University of Iowa, Iowa City, Iowa, USA; ^3^Iowa Institute for Biomedical Imaging, The University of Iowa, Iowa City, Iowa, USA; ^4^Electrical and Computer Engineering, The University of Iowa, Iowa City, Iowa, USA

##### **Correspondence:** Hans J. Johnson (hans-johnson@uiowa.edu) – Electrical and Computer Engineering, The University of Iowa, Iowa City, Iowa, USA

**Introduction**

FreeSurfer[1] is a popular software suite for automatic analysis of MRI data, including subcortical and cortical segmentation, as well as cortical surface reconstruction and correspondence. FreeSurfer's most prominent tool, the recon-all workflow, consists of approximately 170 sequentially run commands in a tcsh shell script that uses approximately 50 unique FreeSurfer tools. The purpose of this project is to reconstruct the recon-all workflow from FreeSurfer's tcsh shell script into an equivalent workflow using Nipype which [2].

The goal of this work is to enhance the efficiency and usability of the workflow by allowing it to take advantage of the increasing availability of high performance computing resources. Nipype also enhances the modularity of the workflow which allows for algorithms from other packages (e.g., ANTS[3] FSL[4] BRAINSTools[5] to be explored, added into the workflow, or take the place of existing processing steps. Therefore, the Nipype environment permits increased collaboration on the recon-all workflow and allows for the limitations of the workflow to be easily addressed.

**Approach**

Nipype interfaces were created for the tools used in the recon-all workflow. These interfaces allow developers to recreate in a Nipype workflow the exact same commands used in the FreeSurfer's tcsh script. The Nipype version of the recon-all workflow was then created by using the Nipype interfaces to connect the FreeSurfer commands in the order necessary. To verify that the new Nipype workflow is equivalent to FreeSurfer's recon-all workflow, both workflows were run on the same set of MRI images on multiple platforms (CentOS 6.4 and Mac OS X) and in a high-performance computing environment. Output surface files were converted to VTK file format, and the output image files were converted to NIFTI file format. The images and surfaces output from FreeSurfer's recon-all workflow were compared to the outputs from Nipype recon-all workflow.

**Results**

**Fig. 13 (abstract A15).**
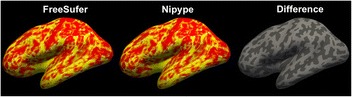
Visualization of the cortical thickness projected onto the left hemisphere's inflated surface for both the FreeSurfer(left) and Nipype workflows as well as the difference the two thicknesses(right). The thickness measurements showed no difference between the workflows

All output images and surfaces from FreeSurfer's recon-all were identical to those of the Nipype workflow that was run on the same operating system (see Fig. 13). During testing on a 16 Core CentOS 6.4 machine with 64GB of memory, FreeSurfer's recon-all workflow completed processing in over 8.9 hours. With multiprocessing, the Nipype workflow achieved identical results and completed processing in under 6 hours when tested on the same machine.

**Conclusions**

The Nipype workflow created in this project was shown to be equivalent to the pre-existing FreeSurfer recon-all workflow. Furthermore, by utilizing Nipype's ability to run commands in parallel, the new workflow reduces the running time of recon-all by over 30% when compared to FreeSurfer's recon-all script which runs commands in a sequential order. The Nipype environment also allows for increased collaboration in further developing the workflow. Future work will involve incorporating more options to the Nipype workflow so that it can function as a complete replacement for the tcsh shell script. The collaborations at the 2015 OHBM BrainHack meeting were instrumental in accomplishing this task. Collaborations with the FreeSurfer, Nipype, and Human Connectome teams allowed members of this project to quickly identify problems and avoid unnecessary failures.

**Availability of supporting data**

The resulting workflow from this project can be found at: https://github.com/nipy/nipype. An example of how to run the workflow on the tutorial data can be found at: https://github.com/nipy/nipype/blob/master/examples/smri_fsreconall.py.

**Competing interests**

None.

**Author’s contributions**

HJJ performed the project and wrote the report. DGE created Nipype interfaces for the FreeSurfer commands, scripted the Nipype workflows, ran the workflow output comparisons, and contributed to the report. IO and RK advised on the project and contributed to the report.

**Acknowledgements**

The authors would like to thank the organizers and attendees of the 2015 OHBM Hackathon. This paper is funded by multiple grants: Huntington's Disease Society of America (Human Biology Project Fellowship), 3D Shape Analysis for Computational Anatomy (R01 EB008171), Neurobiological Predictors of HD (R01 NS040068), Cognitive and Functional Brain Changes in Preclinical HD (R01 NS054893), Algorithms for Functional and Anatomical Brain Analysis (P41 RR015241), Enterprise Storage in a Collaborative Neuroimaging Environment (S10 RR023392), Core 2b HD (U54 EB005149), and Nipype (R03 EB008673).

**References**

1. Fischl B. FreeSurfer. Neuroimage. 2012; 62: 774–781.

2. Gorgolewski K, Burns CD, Madison C, Clark D, Halchenko YO, Waskom ML, Ghosh SS. Nipype: a flexible, lightweight and extensible neuroimaging data processing framework in python. Front Neuroinform. 2011; 5.

3. Avants BB, Tustison N, Song G. Advanced normalization tools (ANTS). Insight J. 2009;

4. Woolrich MW, Jbabdi S, Patenaude B, Chappell M, Makni S, Behrens T, Beckmann C, Jenkinson M, Smith SM. Bayesian analysis of neuroimaging data in FSL. NeuroImage. 2009;

## A16 Facilitating big data meta-analyses for clinical neuroimaging through ENIGMA wrapper scripts

### Erik Kan^1^, Julia Anglin^2^, Michael Borich^3^, Neda Jahanshad^4^, Paul Thompson^4^, Sook-Lei Liew^5^

#### ^1^The Saban Research Institute of Children’s Hospital, Los Angeles, California, USA & Department of Pediatrics of the Keck School of Medicine, University of Southern California, Los Angeles, CA, USA; ^2^Chan Division of Occupational Science and Occupational Therapy, USC, Los Angeles, CA, USA; ^3^Division of Physical Therapy, Department of Rehabilitation Medicine, Emory University School of Medicine, Atlanta, GA, USA; ^4^Imaging Genetics Center, Laboratory of Neuro Imaging, Keck School of Medicine of USC, University of Southern California, Los Angeles, CA, USA; ^5^Chan Division of Occupational Science and Occupational Therapy, Division of Biokinesiology and Physical Therapy, Department of Neurology of the Keck School of Medicine, USC, Los Angeles, CA, USA

##### **Correspondence:** Sook-Lei Liew (sliew@usc.edu) – Chan Division of Occupational Science and Occupational Therapy, Division of Biokinesiology and Physical Therapy, Department of Neurology of the Keck School of Medicine, USC, Los Angeles, CA, USA

**Introduction**

A vast number of clinical disorders may involve changes in brain structure that are correlated with cognitive function and behavior (e.g., depression, schizophrenia, stroke, etc.). Reliably understanding the relationship between specific brain structures and relevant behaviors in worldwide clinical populations could dramatically improve healthcare decisions around the world. For instance, if a reliable relationship between brain structure after stroke and functional motor ability was established, brain imaging could be used to predict prognosis/recovery potential for individual patients. However, high heterogeneity in clinical populations in both individual neuroanatomy and behavioral outcomes make it difficult to develop accurate models of these potentially subtle relationships.

Large neuroimaging studies (n > 10,000) would provide unprecedented power to successfully relate clinical neuroanatomy changes with behavioral measures. While these sample sizes might be difficult for any one individual to collect, the ENIGMA Center for WorldwideMedicine, Imaging, and Genomics has successfully pioneered meta- and mega-analytic methods to accomplish this task. ENIGMA [http://enigma.ini.usc.edu] brings together a global alliance of over 500 international researchers from over 35 countries to pool together neuroimaging data on different disease states in hopes of discovering critical brain-behavior relationships [1,2] Individual investigators with relevant data run ENIGMA analysis protocols on their own data and send back an output folder containing the analysis results to be combined with data from other sites for a meta-analysis. In this way, large sample sizes can be acquired without the hassle of large-scale data transfers or actual neuroimaging data sharing.

ENIGMA protocols were initially developed to harmonize processing methods of imaging researchers around the world and they require a moderate level of familiarity with several programming languages and environments. However, ENIGMA’s recent success has attracted greater interest in collaborative neuroimaging and protocols must be adjusted to allow for all levels of experience, as, the success of this approach depends on individual collaborators running these ENIGMA protocols on their data. Here, we worked on simplifying these protocols so even a novice programmer could use them. In this way, we hope to expand the feasibility of collecting critical clinical data from collaborators who may have less experience with neuroimaging techniques.

**Approach**

The current ENIGMA protocols [http://enigma.ini.usc.edu/protocols/] for structural neuroimaging analyses consist of a number of different word documents with embedded links to different scripts and snippets of code that use R [3], Bash scripting [4], Matlab [5], FSL [6], and Freesurfer [7]. Each step must be run sequentially, costing the user time during the implementation to wait for each step to finish before beginning the next. In addition, the number of different scripts, programming languages and software environments can be challenging for a novice user and introduces numerous instances where the individual may make errors in implementing the code. To address this, we created 3 easy-to-use wrapper scripts that automate the implementation of the ENIGMA protocols for both subcortical and cortical structural MRI analyses (see Table 6). These wrapper scripts reduce over 40 steps down to 3 quick steps for the user. We also created a user-friendly readme file that includes screenshots of the code implementation.

To examine the ease of use and time to implement the new scripts, we tested each of them on 7 users who had different levels of familiarity with programming and neuroimaging (novice users (no programming experience), moderate users (basic-to-intermediate programming experience), and expert users (extensive programming)). To explore additional factors relating to implementation, two of the expert users had to use the scripts in a different environment (e.g., organize the data, install the software, etc.).

**Results/Discussion**

Overall, moderate and expert level users found the scripts extremely easy to implement and required less than 25 minutes to get all three scripts running (excluding the run time of each script; see Table 6 for individual results). The two novice users required greater support to understand basic elements (e.g., what is a terminal), but with support, were able to complete all the steps in less than 1 hour. Finally, the expert users who implemented the scripts on their own environment found the most time-consuming steps to be installing and troubleshooting Freesurfer (e.g., install errors; troubleshooting a conflict between the preset Freesurfer subjects directory and the output directory required by the scripts) and reorganizing data into a format for the scripts (e.g., putting the data into an organized format with main_folder/subject_folder/subject.nii.gz). Once these steps were complete, each expert user reported about 10 minutes for script implementation.

The wrapper scripts made the implementation of the ENIGMA protocols quick and feasible even for novice users. However, there were still three main barriers to participation that required significant time, computational resources, and some expertise: 1) data organization (depending on previous data structure), 2) running freesurfer (~12 hrs/subj), and 3) installation of the required software (e.g., Freesurfer, FSL, R). Future projects may look at ways to streamline these areas for a more seamless user experience in order to facilitate greater sharing of clinical neuroimaging data through ENIGMA.

**Availability of supporting data**

More information about this project can be found at: https://github.com/npnl/ENIGMA-Wrapper-Scripts. A test dataset is available on request; if interested, please email npnl@usc.edu.

**Competing interests**

None.

**Author’s contributions**

EK, JA, and S-LL wrote the wrapper scripts. NJ and PT developed the original ENIGMA protocols and provide guidance/support for ENIGMA working groups. MB, JA, and S-LL tested and edited the wrapper scripts. All authors contributed to the creation of the manuscript.

**Acknowledgements**

Report from 2015 Brainhack Americas (LA). The authors would like to thank the organizers and attendees of Brainhack LA and our testers.

**References**

1. Thompson PM, Andreassen OA, Arias-Vasquez A, Bearden CE, Boedhoe PS, Brouwer RM, Buckner RL, Buitelaar JK, Bulayeva KB, Cannon DM, Cohen RA, Conrod PJ, Dale AM, Deary IJ, Dennis EL, de Reus MA, Desrivieres S, Dima D, Donohoe G, Fisher SE, Fouche JP, Francks C, Frangou S, Franke B, Ganjgahi H, Garavan H, Glahn DC, Grabe HJ, Guadalupe T, Gutman BA, Hashimoto R, Hibar DP, Holl, D, Hoogman M, Pol HE, Hosten N, Jahanshad N, Kelly S, Kochunov P, Kremen WS, Lee PH, Mackey S, Martin NG, Mazoyer B, McDonald C, Medl, SE, Morey RA, Nichols TE, Paus T, Pausova Z, Schmaal L, Schumann G, Shen L, Sisodiya SM, Smit DJ, Smoller JW, Stein DJ, Stein JL, Toro R, Turner JA, van den Heuvel MP, van den Heuvel OL, van Erp TG, van Rooij D, Veltman DJ, Walter H, Wang Y, Wardlaw JM, Whelan CD, Wright MJ, Ye J. ENIGMA and the individual: Predicting factors that affect the brain in 35 countries worldwide. Neuroimage. 2015.

2. Hibar DP, Stein JL, Renteria ME, Arias-Vasquez A, Desrivieres S, Jahanshad N, Toro R, Wittfeld K, Abramovic L, Andersson M, Aribisala BS, Armstrong NJ, Bernard M, Bohlken MM, Boks MP, Bralten J, Brown AA, Chakravarty MM, Chen Q, Ching CR, Cuellar-Partida G, den Braber A, Giddaluru S, Goldman AL, Grimm O, Guadalupe T, Hass J, Woldehawariat G, Holmes AJ, Hoogman M, Janowitz D, Jia T, Kim S, Klein M, Kraemer B, Lee PH, Olde Loohuis LM, Luciano M, Macare C, Mather KA, Mattheisen M, Milaneschi Y, Nho K, Papmeyer M, Ramasamy A, Risacher SL, Roiz-Santianez R, Rose EJ, Salami A, Samann PG, Schmaal L, Schork AJ, Shin J, Strike LT, Teumer A, van Donkelaar MM, van Eijk KR, Walters RK, Westlye LT, Whelan CD, Winkler AM, Zwiers MP, Alhusaini S, Athanasiu L, Ehrlich S, Hakobjan MM, Hartberg CB, Haukvik UK, Heister AJ, Hoehn D, Kasperaviciute D, Liewald DC, Lopez LM, Makkinje RR, Matarin M, Naber MA, McKay DR, Needham M, Nugent AC, Putz B, Royle NA, Shen L, Sprooten E, Trabzuni D, van der Marel SS, van Hulzen KJ, Walton E, Wolf C, Almasy L, Ames D, Arepalli S, Assareh AA, Bastin ME, Brodaty H, Bulayeva KB, Carless MA, Cichon S, Corvin A, Curran JE, Czisch M, de Zubicaray GI, Dillman A, Duggirala R, Dyer TD, Erk S, Fedko IO, Ferrucci L, Foroud TM, Fox PT, Fukunaga M, Gibbs JR, Goring HH, Green RC, Guelfi S, Hansell NK, Hartman CA, Hegenscheid K, Heinz A, Hern,ez DG, Heslenfeld DJ, Hoekstra PJ, Holsboer F, Homuth G, Hottenga JJ, Ikeda M, Jack CR, Jenkinson M, Johnson R, Kanai R, Keil M, Kent JW, Kochunov P, Kwok JB, Lawrie SM, Liu X, Longo DL, McMahon KL, Meisenzahl E, Melle I, Mohnke S, Montgomery GW, Mostert JC, Muhleisen TW, Nalls MA, Nichols TE, Nilsson LG, Nothen MM, Ohi K, Olvera RL, Perez-Iglesias R, Pike GB, Potkin SG, Reinvang I, Reppermund S, Rietschel M, Romanczuk-Seiferth N, Rosen GD, Rujescu D, Schnell K, Schofield PR, Smith C, Steen VM, Sussmann JE, Thalamuthu A, Toga AW, Traynor BJ, Troncoso J, Turner JA, Valdes Hern,ez MC, van ’t Ent D, van der Brug M, van der Wee NJ, van Tol MJ, Veltman DJ, Wassink TH, Westman E, Zielke RH, Zonderman AB, Ashbrook DG, Hager R, Lu L, McMahon FJ, Morris DW, Williams RW, Brunner HG, Buckner RL, Buitelaar JK, Cahn W, Calhoun VD, Cavalleri GL, Crespo-Facorro B, Dale AM, Davies GE, Delanty N, Depondt C, Djurovic S, Drevets WC, Espeseth T, Gollub RL, Ho BC, Hoffmann W, Hosten N, Kahn RS, Le Hellard S, Meyer-Lindenberg A, Muller-Myhsok B, Nauck M, Nyberg L, P,olfo M, Penninx BW, Roffman JL, Sisodiya SM, Smoller JW, van Bokhoven H, van Haren NE, Volzke H, Walter H, Weiner MW, Wen W, White T, Agartz I, Andreassen OA, Blangero J, Boomsma DI, Brouwer RM, Cannon DM, Cookson MR, de Geus EJ, Deary IJ, Donohoe G, Fern,ez G, Fisher SE, Francks C, Glahn DC, Grabe HJ, Gruber O, Hardy J, Hashimoto R, Hulshoff Pol HE, Jonsson EG, Kloszewska I, Lovestone S, Mattay VS, Mecocci P, McDonald C, McIntosh AM, Ophoff RA, Paus T, Pausova Z, Ryten M, Sachdev PS, Saykin AJ, Simmons A, Singleton A, Soininen H, Wardlaw JM, Weale ME, Weinberger DR, Adams HH, Launer LJ, Seiler S, Schmidt R, Chauhan G, Satizabal CL, Becker JT, Yanek L, van der Lee SJ, Ebling M, Fischl B, Longstreth WT, Greve D, Schmidt H, Nyquist P, Vinke LN, van Duijn CM, Xue L, Mazoyer B, Bis JC, Gudnason V, Seshadri S, Ikram MA, Martin NG, Wright MJ, Schumann G, Franke B, Thompson PM, Medl, SE, Weiner M, Aisen P, Petersen R, Jack CR, Jagust W, Trojanowki JQ, Toga AW, Beckett L, Green RC, Saykin AJ, Morris J, Shaw LM, Khachaturian Z, Sorensen G, Carrillo M, Kuller L, Raichle M, Paul S, Davies P, Fillit H, Hefti F, Holtzman D, Mesulman M, Potter W, Snyder P, Schwartz A, Green RC, Montine T, Petersen R, Aisen P, Thomas RG, Donohue M, Walter S, Gessert D, Sather T, Jiminez G, Beckett L, Harvey D, Donohue M, Jack CR, Bernstein M, Fox N, Thompson P, Schuff N, DeCarli C, Borowski B, Gunter J, Senjem M, Vemuri P, Jones D, Kantarci K, Ward C, Jagust W, Koeppe RA, Foster N, Reiman EM, Chen K, Mathis C, L,au S, Morris J, Cairns NJ, Householder E, Taylor-Reinwald L, Trojanowki JQ, Shaw L, Lee VM, Korecka M, Figurski M, Toga AW, Crawford K, Neu S, Saykin AJ, Foroud TM, Potkin S, Shen L, Faber K, Kim S, Nho K, Weiner MW, Thal L, Khachaturian Z, Khachaturian Z, Frank R, Snyder PJ, Weiner MW, Thal L, Buckholtz N, Potter W, Paul S, Albert M, Hsiao J, Kaye J, Quinn J, Lind B, Carter R, Dolen S, Gutman BA, Schneider LS, Pawluczyk S, Beccera M, Teodoro L, Spann BM, Brewer J, V,erswag H, Fleisher A, Heidebrink JL, Lord JL, Petersen R, Mason SS, Albers CS, Knopman D, Johnson K, Doody RS, Villanueva-Meyer J, Chowdhury M, Rountree S, Dang M, Stern Y, Honig LS, Bell KL, Ances B, Morris JC, Carroll M, Leon S, Householder E, Mintun MA, Schneider S, Oliver A, Marson D, Griffith R, Clark D, Geldmacher D, Brockington J, Roberson E, Grossman H, Mitsis E, deToledo-Morrell L, Shah RC, Duara R, Varon D, Greig MT, Roberts P, Albert M, Onyike C, D’Agostino D, Kielb S, Galvin JE, Pogorelec DM, Cerbone B, Michel CA, Rusinek H, de Leon MJ, Glodzik L, De Santi S, Doraiswamy P, Petrella JR, Wong TZ, Arnold SE, Karlawish JH, Wolk D, Smith CD, Jicha G, Hardy P, Sinha P, Oates E, Conrad G, Lopez OL, Oakley M, Simpson DM, Porsteinsson AP, Goldstein BS, Martin K, Makino KM, Ismail M, Br, C, Mulnard RA, Thai G, Mc-Adams-Ortiz C, Womack K, Mathews D, Quiceno M, Diaz-Arrastia R, King R, Weiner M, Martin-Cook K, DeVous M, Levey AI, Lah JJ, Cellar JS, Burns JM, Anderson HS, Swerdlow RH, Apostolova L, Tingus K, Woo E, Silverman DH, Lu PH, Bartzokis G, Graff-Radford NR, Parfitt F, Kendall T, Johnson H, Farlow MR, Hake AM, Matthews BR, Herring S, Hunt C, van Dyck CH, Carson RE, MacAvoy MG, Chertkow H, Bergman H, Hosein C, Black S, Stefanovic B, Caldwell C, Hsiung YR, Feldman H, Mudge B, Assaly M, Kertesz A, Rogers J, Trost D, Bernick C, Munic D, Kerwin D, Mesulam MM, Lipowski K, Wu CK, Johnson N, Sadowsky C, Martinez W, Villena T, Turner RS, Johnson K, Reynolds B, Sperling RA, Johnson KA, Marshall G, Frey M, Yesavage J, Taylor JL, Lane B, Rosen A, Tinklenberg J, Sabbagh MN, Belden CM, Jacobson SA, Sirrel SA, Kowall N, Killiany R, Budson AE, Norbash A, Johnson PL, Obisesan TO, Wolday S, Allard J, Lerner A, Ogrocki P, Hudson L, Fletcher E, Carmichael O, Olichney J, DeCarli C, Kittur S, Borrie M, Lee TY, Bartha R, Johnson S, Asthana S, Carlsson CM, Potkin SG, Preda A, Nguyen D, Tariot P, Fleisher A, Reeder S, Bates V, Capote H, Rainka M, Scharre DW, Kataki M, Adeli A, Zimmerman EA, Celmins D, Brown AD, Pearlson GD, Blank K, Anderson K, Santulli RB, Kitzmiller TJ, Schwartz ES, Sink KM, Williamson JD, Garg P, Watkins F, Ott BR, Querfurth H, Tremont G, Salloway S, Malloy P, Correia S, Rosen HJ, Miller BL, Mintzer J, Spicer K, Bachman D, Finger E, Pasternak S, Rachinsky I, Rogers J, Kertesz A, Drost D, Pomara N, Hern,o R, Sarrael A, Schultz SK, Ponto LL, Shim H, Smith KE, Relkin N, Chaing G, Raudin L, Smith A, Fargher K, Raj BA, Amin N, Becker D, Beiser A, Debette S, DeStefano A, Hofer E, Hofman A, Niessen WJ, Seiler S, Smith A, Tzourio C, Vaidya D, Vernooij MW, Goldstein DB, Heinzen EL, Shianna K, Radtke R, Ottmann R, Albrecht L, Andrew C, Arroyo M, Artiges E, Aydin S, Bach C, Banaschewski T, Barbot A, Barker G, Boddaert N, Bokde A, Bricaud Z, Bromberg U, Bruehl R, Buchel C, Cachia A, Cattrell A, Conrod P, Constant P, Crombag H, Czech K, Dalley J, Decideur B, Desrivieres S, Fadai T, Flor H, Frouin V, Fuchs B, Gallinat J, Garavan H, Bri, FG, Gowl, P, Head K, Heinrichs B, Heinz A, Heym N, Hubner T, Ihlenfeld A, Irel, J, Ittermann B, Ivanov N, Jia T, Jones J, Klaassen A, Lalanne C, Lathrop M, Lanzerath D, Lemaitre H, Ludemann K, Macare C, Mallik C, Mangin JF, Mann K, Mar A, Martinot JL, Massicotte J, Mennigen E, Mesquita de Carvahlo F, Mignon X, Mir,a R, Muller K, Nees F, Nymberg C, Paillere ML, Paus T, Pausova Z, Pena-Oliver Y, Poline JB, Poustka L, Rapp M, Reed L, Robert G, Reuter J, Rietschel M, Ripke S, Ripley T, Robbins T, Rodehacke S, Rogers J, Romanowski A, Ruggeri B, Schilling C, Schmal C, Schmidt D, Schneider S, Schroeder M, Schubert F, Schwartz Y, Smolka M, Sommer W, Spanagel R, Speiser C, Spranger T, Stedman A, Steiner S, Stephens D, Strache N, Strohle A, Struve M, Subramaniam N, Theobald D, Topper L, Vollstaedt-Klein S, Walaszek B, Walter H, Weiss K, Werts H, Whelan R, Williams S, Yacubian J, Ziesch V, Zilbovicius M, Wong CP, Lubbe S, Martinez-Medina L, Kepa A, Fern,es A, Tahmasebi A, Abrahamowicz M, Gaudet D, Leonard G, Perron M, Richer L, Seguin J, Veillette S. Common genetic variants influence human subcortical brain structures. Nature. 2015; 520: 224–229.

3. R Development Core Team. R: A Language and Environment for Statistical Computing. Vienna, Austria: R Foundation for Statistical Computing; 2008..

4. Burtch Ken O. Linux Shell Scripting with Bash. Carmel, Indiana: Sams Publishing; 2004.

5. MATLAB. version 7.10.0 (R2010a). Natick, Massachusetts: The MathWorks Inc.; 2010.

6. Jenkinson M, Beckmann CF, Behrens TE, Woolrich MW, Smith SM. FSL. Neuroimage. 2012; 62: 782–790.

7. Fischl B. FreeSurfer. Neuroimage. 2012; 62: 774–781.

**Table 6 (abstract A16).** User feedback

User Level	EOU	Time	Notes
Novice 1	8	00:35:25	Required walk through support
Novice 2	8	00:52:55	Required support for basic terminal commands only; then was able to complete independently
Moderate 1	3	00:23:45	Required no support
Moderate 2	4	00:22:10	Required no support
Expert 1	3	00:11:34	Required no support
Expert 2 - DE	3	02:00:00	Getting scripts to run took several minutes but reorganizing data and troubleshooting with freesurfer took significant time
Expert 3 – DE	2	01:15:00	Required walk through support

EOU: Ease of Use score (1–10) 1 = easiest, 10 = hardest. Time: the time it took for the user to setup and learn to use the scripts. DE: User’s expertise is with a different computational environment than the one required by the scripts

## A17 A cortical surface-based geodesic distance package for Python

### Daniel S. Margulies, Marcel Falkiewicz, Julia M. Huntenburg

#### Max Planck Research Group for Neuroanatomy & Connectivity, Max Planck Institute for Human Cognitive and Brain Sciences, Leipzig, Germany

##### **Correspondence:** Daniel S. Margulies (margulies@cbs.mpg.de) – Max Planck Research Group for Neuroanatomy & Connectivity, Max Planck Institute for Human Cognitive and Brain Sciences, Leipzig, Germany

**Introduction**

The human cerebral cortex, whether tracing it through phylogeny or ontogeny, emerges through expansion and progressive differentiation into larger and more diverse areas. While current methodologies address this analytically by characterizing local cortical expansion in the form of surface area [1] several lines of research have proposed that the cortex in fact expands along trajectories from primordial anchor areas [2,3] and furthermore, that the distance along the cortical surface is informative regarding cortical differentiation [4]. We sought to investigate the geometric relationships that arise in the cortex based on expansion from such origin points. Towards this aim, we developed a Python package for measuring the geodesic distance along the cortical surface that restricts shortest paths from passing through nodes of non-cortical areas such as the non-cortical portions of the surface mesh described as the “medial wall’.

**Approach**

The calculation of geodesic distance along a mesh surface is based in the cumulative distance of the shortest path between two points. The first challenge that arises is the sensitivity of the calculation to the resolution of the mesh: the coarser mesh, the longer the shortest path may be, as the distance becomes progressively less direct. This problem has been previously addressed and subsequently implemented in the Python package gdist [https://pypi.python.org/pypi/gdist/], which calculates the exact geodesic distance along a mesh by subdividing the shortest path until a straight line along the cortex is approximated [5]

The second challenge, for which there was no prefabricated solution, was ensuring that the shortest path only traverses territory within the cortex proper, avoiding shortcuts through non-cortical areas included in the surface mesh — most prominently, the non-cortical portions along the medial wall. Were the shortest paths between two nodes to traverse non-cortical regions, the distance between nodes would be artificially decreased, which would have artifactual impact on the interpretation of results. This concern would be especially relevent to the ‘zones analysis’ described below, where the boundaries between regions would be altered. It was therefore necessary to remove mesh nodes prior to calculating the exact geodesic, which requires reconstructing the mesh and assigning the respective new node indices for any seed regions-of-interest.

Finally, to facilitate applications to neuroscience research questions, we enabled the loading and visualization of data from commonly used formats such as FreeSurfer and the Human Connectome Project (HCP). A Nipype pipeline for group-level batch processing has also been made available [6]. The pipeline is wrapped in a command-line interface and allows for straightforward distance calculations of entire FreeSurfer-preprocessed datasets. Group-level data are stored as CSV files for each requested mesh resolution, source label and hemisphere, facilitating further statistical analyses.

**Results**

**Fig. 14 (abstract A17).**
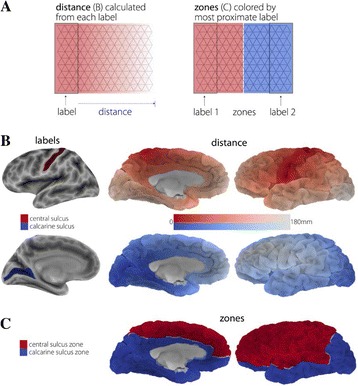
**a** Schematic illustrating the distance (**b**) and zone (**c**) analyses. **b** FreeSurfer labels from the central and calcarine sulci depicted on the individual inflated surface (left), and the exact geodesic distance from the two labels presented on an individual pial surface (right). **c** Zones delineated based on proximity to the central (red) or calcarine (blue) sulci

The resultant package, SurfDist, achieves the aforementioned goals of faciliating the calculation of exact geodesic distance on the cortical surface. We present here the distance measures from the central and calcarine sulci labels on the FreeSurfer native surfaces (Fig. 14b). The distance measure provides a means to parcellate the cortex using the surface geometry. Towards that aim, we also implement a ‘zones analysis’, which constructs a Voronoi diagram, establishing partitions based on the greater proximity to a set of label nodes (Fig. 14c).

Surface rendering of the results draws from plotting functions as implemented in Nilearn [7] and exclusively relies on the common library matplotlib to minimize dependencies. The visualization applies sensible defaults but can flexibly be adapted to different views, colormaps and thresholds as well as shadowing using a sulcal depth map.

**Conclusions**

The SurfDist package is designed to enable investigation of intrinisic geometric properties of the cerebral cortex based on geodesic distance measures. Towards the aim of enabling applications specific to neuroimaging-based research question, we have designed the package to facilitate analysis and visualization of geodesic distance metrics using standard cortical surface meshes.

**Availability of supporting data**

More information about this project can be found at: http://github.com/margulies/surfdist

**Competing interests**

None.

**Author’s contributions**

DSM, MF, and JMH wrote the software and report.

**Acknowledgements**

Report from 2015 Brainhack Americas (MX). The authors would like to thank the organizers and attendees of Brainhack MX. The visualization functions were originally developed during the Nilearn coding sprint 2015 in Paris, for which we would also like to thank the organizers and participants of this event.

**References**

1. Winkler Anderson M, Sabuncu Mert R, Yeo BT Thomas, Fischl Bruce, Greve Douglas N, Kochunov Peter, Nichols Thomas E, Blangero John, Glahn David C. Measuring and comparing brain cortical surface area and other areal quantities. Neuroimage. 2012; 61: 1428–1443.

2. Sanides Friedrich. Comparative architectonics of the neocortex of mammals and their evolutionary interpretation. Ann N Y Acad Sci. 1969; 167: 404–423.

3. Buckner R,y L, Krienen Fenna M. The evolution of distributed association networks in the human brain. Trends Cogn Sci. 2013; 17: 648–665.

4. Wagstyl Konrad, Ronan Lisa, Goodyer Ian M, Fletcher Paul C. Cortical thickness gradients in structural hierarchies. Neuroimage. 2015; 111: 241–250.

5. Mitchell Joseph S B, Mount David M, Papadimitriou Christos H. The Discrete Geodesic Problem. SIAM J Comput. 1987; 16: 647–668.

6. Gorgolewski Krzysztof, Burns Christopher D, Madison Cindee, Clark Dav, Halchenko Yaroslav O. Nipype: a flexible, lightweight and extensible neuroimaging data processing framework in Python. Frontiers in Neuroinformatics. 2011; 5.

7. Abraham Alex,re, Pedregosa Fabian, Eickenberg Michael, Gervais Philippe, Mueller Andreas, Kossaifi Jean, Gramfort Alex,re, Thirion Bertr, Varoquaux Gaël. Machine learning for neuroimaging with scikit-learn. Frontiers in Neuroinformatics. 2014; 8: 1–10.

## A18 Sharing data in the cloud

### David O’Connor^1,2^, Daniel J. Clark^2^, Michael P. Milham^1,2^, R. Cameron Craddock^1,2^

#### ^1^Center for Biomedical Imaging and Neuromodulation, Nathan Kline Institute for Psychiatric Research, Orangeburg, NY, USA; ^2^Center for the Developing Brain, Child Mind Institute, New York, NY, USA

##### **Correspondence:** David O’Connor (david.oconnor@childmind.org) – Center for Biomedical Imaging and Neuromodulation, Nathan Kline Institute for Psychiatric Research, Orangeburg, NY, USA

**Introduction**

Cloud computing resources, such as Amazon Web Services (AWS) [http://aws.amazon.com], provide pay-as-you-go access to high-performance computer resources and dependable data storage solutions for performing large scale analyses of neuroimaging data [1]. These are particularly attractive for researchers at small universities and in developing countries who lack the wherewithal to maintain their own high performance computing systems. The objective of this project is to upload data from the 1000 Functional Connectomes Project (FCP) [2] and International Neuroimaging Datasharing Initiatives (INDI) [3] grass-roots data sharing initiatives into a Public S3 Bucket that has been generously provided by AWS. This will make the data more quickly accessible for AWS-based analysis of these data, but will also improve the speed and availability of access to this data for analyses performed outside of the cloud. To begin with, we focused on the following collections:

- The autism brain imaging data exchange *(ABIDE)* consists of structural MRI and resting state functional MRI from 1113 individuals (164 F, 948 M, 6–64 years old, 539 with autism spectrum disorders, 573 typical controls) aggregated from 20 different studies [4]
- The *ADHD-200* contains structural MRI and resting state functional MRI from 973 individuals (352 F, 594 M, 7–21 years old, 362 with attention deficit hyperactivity disorder (ADHD), 585 typically developing controls) collected from 8 sites [5]
- The *Consortium for Reliability and Reproducibility (CoRR)* consists of 3,357 structural MRI, 5,093 resting state fMRI, 1,302 diffusion MRI, and 300 cerebral blood flow scans from 1629 subjects (673 F, 956 M, 6–84 years old, all typical controls) acquired in a variety of test-retest designs at 35 sites [6]
- The *Enhanced Nathan Kline Institute - Rockland Sample (ENKI-RS)* consists of structural MRI, resting state functional MRI, diffusion MRI, cerebral blood flow, and a variety of task functional MRI scans and deep phenotyping on over 700 participants from across the lifespan and a variety of phenotypes acquired at a single site [7] The acquisition of this collection is ongoing.
- The *Addiction Connectome Preprocessed Initiative (ACPI)* [http://fcon_1000.projects.nitrc.org/indi/ACPI/html/index.html] consists of 216 structural MRI and 252 functional MRI from 192 subjects (44 F, 148 M, 18–50 years old) from three datasets generated by NIDA investigators.

**Approach**

Data for the ADHD-200, ABIDE, CoRR, and Rockland Sample data collections are currently downloadable from NITRC [http://fcon_1000.projects.nitrc.org/] as a series of large (>2GB) tar files. The process of uploading the data involved downloading and extracting the data from these tar files, organizing the individual images to the standardized INDI format [http://fcon_1000.projects.nitrc.org/indi/indi_data_contribution_guide.pdf] and then uploading the data to S3. We developed a S3 upload script in python using the Boto AWS software development kit [https://aws.amazon.com/sdk-for-python/] to facilitate this process. We also developed a download script in python that provides basic query functionality for selecting the data to download from a spreadsheet describing the data.

**Results**

**Fig. 15 (abstract A18).**
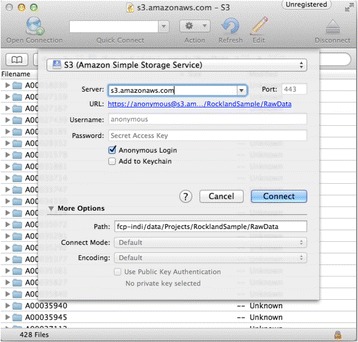
Connecting to the data repository

The entirety of the CoRR, ABIDE, ACPI, and ADHD-200 data collections and ENKIRS data for 427 individuals were uploaded during the OHBM Hackathon event. The data are available as individual files to make it easily indexable by database infrastructures such as COINS [8] LORIS [9] and others. Additionally, this makes it easy for the users to download just the data that they want. The data in the bucket can be browsed and downloaded using a GUI based S3 file transfer software such as Cyberduck [http://cyberduck.io] (see Fig. 14), or using the Boto Python library [https://github.com/FCP-INDI/INDI-Tools]. One can connect to the bucket using the configuration shown in Fig. 15. The data is structured as follows: bucketname/data/Projects/ProjectName/DataType. For example you can access raw data from the ENKI-RS, as shown in Fig. 15, by specifying the following path in CyberDuck: https://s3.amazon.com/fcp-indi/data/Projects/RocklandSample/RawData

**Conclusions**

Uploading data shared through the FCP and INDI initiatives improves its accessibility for cloud-based and local computation. Future efforts for this project will include uploading the remainder of the FCP and INDI data and organizing the data in the new brain imaging data structure (BIDS) format [10].

**Availability of supporting data**

More information about this project can be found at: https://github.com/DaveOC90/INDI-Organization-Scripts

**Competing interests**

None.

**Author’s contributions**

DO performed quality control, and uploaded the data. DJC wrote code to interact with AWS, preprocessed and uploaded data. MPM and RCC lead the data collection and sharing projects. All of the authors contributed to writing the project report.

**Acknowledgements**

Report from 2015 OHBM Hackathon (HI). The authors would like to thank the organizers and attendees of the OHBM Brainhack in Hawaii. This project was made possible by the S3 public bucket generously provided by Amazon Web Services.

**References**

1. Clark Daniel, Haselgrove Christian, Kennedy David N, Liu Zhizhong, Milham Michael, Petrosyan Petros, Torgerson Carinna, Van Horn John, Craddock Cameron. Harnessing cloud computing for high capacity analysis of neuroimaging data from NDAR. Frontiers in Neuroscience.

2. Biswal BB, Mennes M, Zuo XN, Gohel S, Kelly C, Smith SM, Beckmann CF, Adelstein JS, Buckner RL, Colcombe S, Dogonowski AM, Ernst M, Fair D, Hampson M, Hoptman MJ, Hyde JS, Kiviniemi VJ, Kotter R, Li SJ, Lin CP, Lowe MJ, Mackay C, Madden DJ, Madsen KH, Margulies DS, Mayberg HS, McMahon K, Monk CS, Mostofsky SH, Nagel BJ, Pekar JJ, Peltier SJ, Petersen SE, Riedl V, Rombouts SA, Rypma B, Schlaggar BL, Schmidt S, Seidler RD, Siegle GJ, Sorg C, Teng GJ, Veijola J, Villringer A, Walter M, Wang L, Weng XC, Whitfield-Gabrieli S, Williamson P, Windischberger C, Zang YF, Zhang HY, Castellanos FX, Milham MP. Toward discovery science of human brain function. Proc Natl Acad Sci USA. 2010; 107: 4734–4739.

3. Mennes M, Biswal BB, Castellanos FX, Milham MP. Making data sharing work: the FCP/INDI experience. Neuroimage. 2013; 82: 683–691.

4. Di Martino A, Yan CG, Li Q, Denio E, Castellanos FX, Alaerts K, Anderson JS, Assaf M, Bookheimer SY, Dapretto M, Deen B, Delmonte S, Dinstein I, Ertl-Wagner B, Fair DA, Gallagher L, Kennedy DP, Keown CL, Keysers C, Lainhart JE, Lord C, Luna B, Menon V, Minshew NJ, Monk CS, Mueller S, Muller RA, Nebel MB, Nigg JT, O’Hearn K, Pelphrey KA, Peltier SJ, Rudie JD, Sunaert S, Thioux M, Tyszka JM, Uddin LQ, Verhoeven JS, Wenderoth N, Wiggins JL, Mostofsky SH, Milham MP. The autism brain imaging data exchange: towards a large-scale evaluation of the intrinsic brain architecture in autism. Mol Psychiatry. 2014; 19: 659–667.

5. Milham Michael P, Fair Damien, Mennes Maarten, Mostofsky Stewart H. The adhd-200 consortium: a model to advance the translational potential of neuroimaging in clinical neuroscience. Frontiers in Systems Neuroscience. 2012; 6.

6. Zuo XN, Anderson JS, Bellec P, Birn RM, Biswal BB, Blautzik J, Breitner JC, Buckner RL, Calhoun VD, Castellanos FX, Chen A, Chen B, Chen J, Chen X, Colcombe SJ, Courtney W, Craddock RC, Di Martino A, Dong HM, Fu X, Gong Q, Gorgolewski KJ, Han Y, He Y, He Y, Ho E, Holmes A, Hou XH, Huckins J, Jiang T, Jiang Y, Kelley W, Kelly C, King M, LaConte SM, Lainhart JE, Lei X, Li HJ, Li K, Li K, Lin Q, Liu D, Liu J, Liu X, Liu Y, Lu G, Lu J, Luna B, Luo J, Lurie D, Mao Y, Margulies DS, Mayer AR, Meindl T, Meyer, ME, Nan W, Nielsen JA, O’Connor D, Paulsen D, Prabhakaran V, Qi Z, Qiu J, Shao C, Shehzad Z, Tang W, Villringer A, Wang H, Wang K, Wei D, Wei GX, Weng XC, Wu X, Xu T, Yang N, Yang Z, Zang YF, Zhang L, Zhang Q, Zhang Z, Zhang Z, Zhao K, Zhen Z, Zhou Y, Zhu XT, Milham MP. An open science resource for establishing reliability and reproducibility in functional connectomics. Sci Data. 2014; 1.

7. Nooner KB, Colcombe SJ, Tobe RH, Mennes M, Benedict MM, Moreno AL, Panek LJ, Brown S, Zavitz ST, Li Q, Sikka S, Gutman D, Bangaru S, Schlachter RT, Kamiel SM, Anwar AR, Hinz CM, Kaplan MS, Rachlin AB, Adelsberg S, Cheung B, Khanuja R, Yan C, Craddock CC, Calhoun V, Courtney W, King M, Wood D, Cox CL, Kelly AM, Di Martino A, Petkova E, Reiss PT, Duan N, Thomsen D, Biswal B, Coffey B, Hoptman MJ, Javitt DC, Pomara N, Sidtis JJ, Koplewicz HS, Castellanos FX, Leventhal BL, Milham MP. The NKI-Rockland Sample: A Model for Accelerating the Pace of Discovery Science in Psychiatry. Front Neurosci. 2012; 6.

8. L,is D, Courtney W, Dieringer C, Kelly R, King M, Miller B, Wang R, Wood D, Turner JA, Calhoun VD. COINS Data Exchange: An open platform for compiling, curating, and disseminating neuroimaging data. Neuroimage. 2016; 124: 1084–1088.

9. Das S. LORIS: a web-based data management system for multi-center studies. FrontNeuroinform. 2011.

10. Gorgolewski Krzysztof Jacek, Auer Tibor, Calhoun Vince D, Craddock RCameron, Das Samir, Duff Eugene P, Fl,in Guillaume, Ghosh Satrajit S, Glatard Tristan, Halchenko Yaroslav O, H,werker Daniel A, Hanke Michael, Keator David, Li Xiangrui, Michael Zachary, Maumet Camille, Nichols BNolan, Nichols Thomas E, Poline Jean-Baptiste, Rokem Ariel, Schaefer Gunnar, Sochat Vanessa, Turner Jessica A, Varoquaux Ga“el, Poldrack Russell A. The Brain Imaging Data Structure: a standard for organizing and describing outputs of neuroimaging experiments. bioRxiv. 2015.

## A19 Detecting task-based fMRI compliance using plan abandonment techniques

### Ramon Fraga Pereira, Anibal Sólon Heinsfeld, Alexandre Rosa Franco, Augusto Buchweitz, Felipe Meneguzzi

#### PUCRS, Porto Alegre, Rio Grande do Sul, Brazil

##### **Correspondence:** Ramon Fraga Pereira (ramon.pereira@acad.pucrs.br) – PUCRS, Porto Alegre, Rio Grande do Sul, Brazil

**Introduction**

Task-based fMRI is a powerful approach to understand brain processes for a certain task. However, fMRI images are usually preprocessed hours, days or even months after the scan. During the functional image preprocessing stage, defects in images are detected and, in some cases, cannot be corrected. For example, technical problems with the scanner or lack of collaboration from the subject to perform the given tasks. For these cases it is necessary to realize a new scan. In order to mitigate lost scans due to patient non-compliance, we need an approach to detect such non-compliance during the scan.

**Approach**

In this Brainhack project, we aim to detect if a subject is following the given task and provide an almost real-time feedback to the researchers to make a decision during the exam if the subject is not collaborating. This is necessary to be performed in order to avoid loss of data, in which the images are typically processed and quality assessed at another day. We will focus on task where there are no button responses from the subject, hence relying solely in the BOLD signal if the subject is collaborating. To do so, we use plan abandonment techniques [1] a sub-area of Artificial Intelligence. For a given fMRI paradigm, a plan should be created and compared with the subject’s brain activation during the scan using recognition methods. To use plan abandonment techniques, we need to discretize and formalize the fMRI and construct a expected plan based on the hypothesized paradigm using this formalization. To evaluate the compliance with a specific paradigm, we aim to use real-time fMRI methods to retrieve BOLD signals of brain regions that are supposed to be active in a particular time range. In order to tolerate fluctuations of the BOLD signal, we aim to use the methods that detect non-compliance using a threshold from the expected activation.By doing so, it is possible to detect if a subject is following the paradigm given a specific stimulus type, such as visual or auditory stimulus. The brain state of each stimulus type will be mapped based on atlas from the literature. For example, to cover motor activations, Brodmann area 4 will be mapped with a state *motor_actv*. Thus, for a paradigm that works with motor tasks, the plan must contain *motor_actv* for the given time that the task occurs.

**Discussion**

The formalization of brain states strongly depends on the discretization of specific region states, which might vary from subject to subject. In order to normalize the signals, a previous tuning phase is required with simple paradigms, depending on which paradigm will be executed. During the scan, an online normalization must be made to a standard space, such as the MNI brain space. This real-time processing is required to map expected active regions to the previously selected brain areas from an atlas.

The usage of real-time fMRI methods aggregates to our approach since the tuning and pursuance recognition can be made during the exam. Such real-time fMRI methods can also monitor movements during the scan in order to identify if there is too much subject movement. In the case of fMRI paradigm abandonment, the paradigm can be adapted to induce or interest the subject in a way that the subject proceeds with its tasks, using methods such as demonstrated by [2]. Neurofeedback can be used to sustain the subject’s interest by letting the paradigm be more challenging, requiring more attention and collaboration from the patient, such as the paradigm from [3].

**Conclusions**

This project is in its initial phase. Real-time fMRI methods are being tested, using AFNI’s provided tools. In order to use plan abandonment techniques, the next step is to formalize basic stimuli types based on mapped regions. By using these formalizations, paradigms can be converted to a problem of plan abandonment and it becomes possible to evaluate the participation of a subject during the scan.

**Availability of supporting data**

More information about this project can be found at: https://github.com/brainhack-poa/fmri-plan-recongnition.

**Competing interests**

None.

**Author’s contributions**

RFP and FM develop the project, and RFP, ASH, FM, ARF, and AB wrote the report.

**Acknowledgements**

Report from 2015 Brainhack Americas (MX). The authors would like to thank the organizers and attendees of Brainhack MX and the developers of AFNI.

**References**

1. Sukthankar Gita, Goldman Robert P, Geib Christopher, Pynadath David V, Bui Hung. Plan, Activity, and Intent Recognition: Theory and Practice. Burlington, Massachusetts: Morgan Kaufmann; 2014. p. 82–88.

2. Lee Dongha, Park Bumhee, Jang Changwon, Park Hae-Jeong. Decoding brain states using functional magnetic resonance imaging. Biomedical Engineering Letters. 2011; 1: 82–88.

3. deBettencourt MT, Cohen JD, Lee RF, Norman KA, Turk-Browne NB. Closed-loop training of attention with real-time brain imaging.. Nature Neuroscience. 2015; 18: 470–475.

## A20 Self-Organization and Brain Function

### Jörg P. Pfannmöller^1^, Rickson C. Mesquita^2^, Luis C.T. Herrera^2^, Daniela Dentico^3^

#### ^1^Functional Imaging Unit, Center for Diagnostic Radiology, University Medicine Greifswald, Greifswald, Germany; ^2^Institute of Physics, University of Campinas, Campinas, Brazil; ^3^Waisman Center, University of Wisconsin, Madison, Wisconsin, USA

##### **Correspondence:** J. P. Pfannmoller (pfannmoelj@uni-greifswald.de) – Functional Imaging Unit, Center for Diagnostic Radiology, University Medicine Greifswald, Greifswald, Germany

**Introduction**

Self-organization is a fundamental property of complex systems, describing the order spontaneously arising by the local interactions of the system components not mediated by top-down inputs. Though, self-organizing systems typically possess a large number of components and exhibit complex dynamics, their evolution is deterministic and governed by a small number of order parameters. This property was used to model the self-organization of the ocular dominance columns of the striate cortex in patterns of neighboring stripes [1] which respond preferentially to inputs from the left or the right eye. In this model the self-organization across ocular dominance and orientation preference layers was coupled, were both layers were modeled with the Swift-Hohenberg eq. [2] We reduce the model complexity by including only the cortical dominance layer and investigate the parameter dependency of the self-organization with a Matlab implementation.

**Approach**

The Swift-Hohenberg eq. [2] was used to model the self-organization of the ocular dominance columns. There are two order parameters in this equation, the first one determines the spatial wavelength (λ) of the stripes and the second one the branchiness (ε) of the pattern. is the Laplace operator.

1 $$ {\partial}_t\psi \left(x,y,t\right)=\left[\varepsilon -{\left(\Delta +\frac{4{\pi}^2}{\lambda^2}\right)}^2\right]\cdotp \psi -{\psi}^3 $$

The algorithm used to generate the results has been modified from an open source script [http://nile.physics.ncsu.edu/hon292a-f08/]. The Swift-Hohenberg equation was solved by applying periodic boundary conditions after a Fourier transform to k space, which simplifies the computation of the solution.

**Results**

**Fig. 16 (abstract A20).**
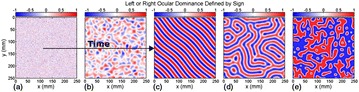
Occular Dominance

Figures 16 (a), (b) and (c) shows the temporal evolution of the solution to the Swift-Hohenberg equation for random initial conditions (a), constant ε and time increasing from (a) to (c). In (c), (d) and (e) three solutions with different ε are shown. The branchiness increases with ε from (c) to (e). The wavelength (λ) was set to the same value in all figures and the pattern in (d) is similar to the ocular dominance layers found in the visual cortex.

**Conclusions**

A simple model suffices to study basic properties of ocular dominance self-organization. Possibly, a combination of models for self-organization in neighboring cortical layers would allow to investigate even higher organizational principles of the cortex [1] e.g.~the coordination between ocular dominance layers, orientation layers, and cytochrome oxidase.

**Availability of supporting data**

More information about this project can be found at: http://brainhack.org/self-organization-and-brain-function. Further data and files supporting this project are hosted in the GigaScience repository: https://github.com/Brainhack-Proceedings-2015/Pfan_HBM_SOBF.

**Competing interests**

None.

**Author’s contributions**

JPP, RCM, LCTH, and DD performed the project and wrote the report.

**Acknowledgements**

The authors would like to thank the organizers and attendees of the 2015 OHBM Hackathon. This work was supported by generous donations from individuals to the Center for Investigating Healthy Minds, founded and led by Dr.~Richard J. Davidson.

**References**

1. Reichl L, Heide D, L"owel S, Crowley JC, Kaschube M, Wolf F. Coordinated Optimization of Visual Cortical Maps (I) Symmetry-based Analysis. PLoS Comput Biol. 2012; 8: e1002466.

2. Hohenberg PC, Swift JB. Effects of additive noise at the onset of Rayleigh-B'enard convection. Phys Rev A. 1992; 46: 4773–4785.

## A21 The Neuroimaging Data Model (NIDM) API

### Vanessa Sochat^1^, B Nolan Nichols^2,3^

#### ^1^Program in Biomedical Informatics, Stanford University, Stanford, California, USA; ^2^SRI International, Menlo Park, CA, USA; ^3^Department of Psychiatry and Behavioral Sciences, Stanford University, Stanford, CA, USA

##### **Correspondence:** Vanessa Sochat (vsochat@stanford.edu) – Program in Biomedical Informatics, Stanford University, Stanford, California, USA

**Introduction**

Sharing of brain research can be aided by the Neuroimaging Data Model (NIDM) [1–3]. NIDM provides a community-based framework for developing data exchange standards that describe the primary observations, computational workflows, and derived results of neuroimaging studies [4]. For example, a researcher sharing a statistical brain map could include with the brain map a data structure, “NIDM Results”, that contains complete information about the parameters used to generate the result, significant coordinate points in the brain map paired with test criteria, along with other meta-data exported from the software that generated it. This additional information cannot be represented in the brain map itself, and provides a complete description of the result that can be compared to other results, or used to reproduce it.

While work is underway to integrate NIDM into the software used by the human brain mapping community, only low-level tools are currently available to access and query NIDM documents that rely on a graph-based representation called the Resource Description Framework (RDF) [5]. Further, technologies like RDF and the corresponding query language, SPARQL [6] pose a steep learning curve for users of standard Web development workflows. With the recent migration of tools for neuroimaging meta analysis [7, 8], sharing [9–17], and visualization [18–20] into the Web browser, Web developers will be incentivized by the ability to easily integrate brain data into Web applications using familiar languages and formats. The goal of this Brainhack project was to develop infrastructure to serve NIDM documents and queries using an API with a syntax that allows for the easy development of Web-based tools for the neuroimaging community. These tools are publicly available on Github (RRID:SCR_002630) for the API [https://github.com/incf-nidash/nidm-api] and queries [https://github.com/incf-nidash/nidm-query], along with complete documentation [https://nidm-api.readthedocs.org].

**Approach**

**Fig. 17 (abstract A21).**
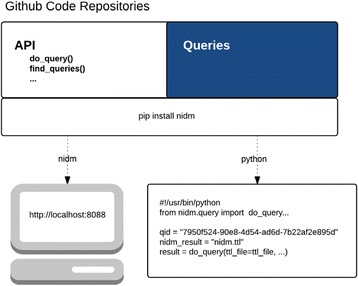
The nidm-api (nidm) provides programmatic access to queries in the nidm-query repository, including RESTful access (left panel) and access from python applications (right panel)

The nidm-api [21] is a RESTful API and Web application that provides a simplified view of NIDM documents using formats (e.g., JavaScript Object Notation (JSON) [22] that are accessible to Web developers and researchers without expertise in Linked Open Data (LOD) technologies. This project includes two components. First, the nidm-api [https://github.com/incf-nidash/nidm-api] is a Python-based executable that works both as a command-line tool to run queries over NIDM documents, as well as to serve a RESTful API to allow a local or cloud-based server to execute queries on documents accessible by URL. Second, nidm-query [https://github.com/incf-nidash/nidm-query] is a repository of SPARQL queries that the nidm-api application dynamically downloads, validates, and serves upon starting the application. This strategy means that NIDM developers can collaboratively construct SPARQL queries without requiring Web developers to gain expertise in LOD technology. The nidm-api, along with serving the queries, also provides a graphical Web interfaces to contribute new queries to the shared repository. Because the nidm-api is a Python Flask [23] application, it can be used both as an executable to serve the API [24] and contains a set of functions that can be integrated into other Python-based frameworks [25] or cloud platforms that provide Python accessibility [26, 27]. A schematic of the tool is provided in (Fig. 17).

**Results**

**Using the API**

Installation produces an executable, *nidm* that downloads, validates, and provides a summary of available queries in the nidm-query repository. A query can be further investigated by selecting its unique identifier: http://localhost:8088/api/7950f524-90e8-4d54-ad6d-7b22af2e895d and can then be executed in a RESTful fashion by including a variable to point to a local path or URL of a NIDM document: http://localhost:8088/api/query/7950f524-90e8-4d54-ad6d-7b22af2e895d?ttl=/home/nidm.ttl.

The API then runs the query over the document, and returns the result to the user in JSON. The same functionality can be achieved on the command line [http://nidm-api.readthedocs.org/en/latest/getting-started.html#integration-into-python], supporting direct integration into server-based Python applications.

**Generating new queries**

Researchers familiar with LOD can run the application in the same fashion, and go to a URL in their local browser:

http://localhost:8088/query/new which reveals an interface [http://nidm-api.readthedocs.org/en/latest/development.html#web-query-generator] to generate new queries. The web interface asks for a set of variables [http://nidm-api.readthedocs.org/en/latest/development.html#fields] that are necessary for the nidm-api to serve the query. The query can be previewed, and then downloaded as a JSON object that can be submitted to the nidm-query repository and added to the application.

**Applications using NIDM**

**Fig. 18 (abstract A21).**
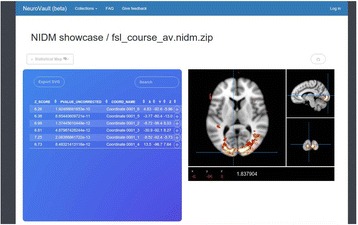
The nidm-viewer in the NeuroVault database queries NIDM Results objects to generate an interactive table and statistical brain map

As an example of the type of Web applications that can be built with the NIDM API, the NIDM Results object model [28] was recently integrated into the NeuroVault [http://www.neurovault.org] database, meaning that neuroimaging researchers can export results pertaining to statistical brain maps from common software [29] into NeuroVault. A nidm-viewer [https://github.com/vsoch/nidmviewer] that runs queries over the nidm-results can then parse the coordinates and statistical parameters associated with significant locations of activations to be rendered in a table alongside a visualization of the brain map itself (Fig. 18 and example [http://neurovault.org/collections/877/fsl_course_av.nidm]). The raw data and parameters of the analysis are thus immediately available for sharing and publication, programatically accessible, and viewed from any web browser.

**Conclusions**

By providing tools to integrate the NIDM standard into modern web technology, NIDM can be more easily deployed into applications to empower neuroimaging researchers to explore and synthesize results, workflows, and experiments. This application will be extended to return more modern and desired outputs such as images and interactive graphs [30] and additional functionality will be added as the NIDM experiment, workflows, and results standards are further developed. The software and queries are both publicly available [https://github.com/incf-nidash] and open to contributions.

**Availability of Supporting Data**

More information about this project can be found at: http://nidm-api.readthedocs.org. Further data and files supporting this project are hosted in the *INCF NIDASH* repositories https://github.com/incf-nidash/nidm-api and https://github.com/incf-nidash/nidm-query.

**Competing interests**

None.

**Author’s contributions**

VS and NN wrote the software and wrote the report.

**Acknowledgements**

Report from 2015 Brainhack Americas (MX). The authors would like to thank the INCF Neuroimaging Data Sharing Task Force, organizers and attendees of Brainhack MX, along with David Keator for helpful edits to the manuscript. VS is supported by a William R. Hewlett Stanford Graduate Fellowship and a National Science Foundation Fellowship. NN is supported by NIH NIAAA and OD (NCANDA Data Analysis Component, NIH 1 U01 AA021697; BD2K Supplement, NIH 1 U01 AA021697-04S1).

**References**

1. Keator D B, Helmer K, Steffener J, Turner J A, Van Erp T G M, Gadde S, Ashish N, Burns G A, Nichols B N. Towards structured sharing of raw and derived neuroimaging data across existing resources. Neuroimage. 2013; 82: 647–661.

2. Neuroimaging Data Model Overview (NIDM-Overview). http://nidm.nidash.org/specs/nidm-overview.html. Accessed August 17, 2016.

3. NIDM Specifications - Neuroimaging Data Model. http://nidm.nidash.org/. Accessed August 17, 2016.

4. RDF - Semantic Web Standards. http://www.w3.org/RDF/. Accessed August 17, 2016.

5. SPARQL Query Language for RDF. http://www.w3.org/TR/rdf-sparql-query/. Accessed August 17, 2016.

6. Yarkoni Tal, Poldrack Russell A, Nichols Thomas E, Van Essen David C, Wager Tor D. Large-scale automated synthesis of human functional neuroimaging data. Nat Methods. 2011; 8: 665–670.

7. Reid Andrew T, Bzdok Danilo, Genon Sarah, Langner Robert, M“uller Veronika I, Eickhoff Claudia R, Hoffstaedter Felix, Cieslik Edna-Clarisse, Fox Peter T, Laird Angela R, Amunts Katrin, Eickhoff Simon B. ANIMA: A data-sharing initiative for neuroimaging meta-analyses. Neuroimage. 2015.

8. Crawford Karen L, Neu Scott C, Toga Arthur W. The Image and Data Archive at the Laboratory of Neuro Imaging. Neuroimage. 2016; 124: 1080–1083.

9. L,is Drew, Courtney William, Dieringer Christopher, Kelly Ross, King Margaret, Miller Brittny, Wang Runtang, Wood Dylan, Turner Jessica A, Calhoun Vince D. COINS Data Exchange: An open platform for compiling, curating, and disseminating neuroimaging data. Neuroimage. 2016; 124: 1084–1088.

10. Book Gregory A, Stevens Michael C, Assaf Michal, Glahn David C, Pearlson Godfrey D. Neuroimaging data sharing on the neuroinformatics database platform. Neuroimage. 2016; 124: 1089–1092.

11. Herrick Rick, Horton William, Olsen Timothy, McKay Michael, Archie Kevin A, Marcus Daniel S. XNAT Central: Open sourcing imaging research data. Neuroimage. 2016; 124: 1093–1096.

12. Hodge Michael R, Horton William, Brown Timothy, Herrick Rick, Olsen Timothy, Hileman Michael E, McKay Michael, Archie Kevin A, Cler Eileen, Harms Michael P, Burgess Gregory C, Glasser Matthew F, Elam Jennifer S, Curtiss S,ra W, Barch Deanna M, Oostenveld Robert, Larson-Prior Linda J, Ugurbil Kamil, Van Essen David C, Marcus Daniel S. ConnectomeDB-Sharing human brain connectivity data. Neuroimage. 2016; 124: 1102–1107.

13. Jernigan Terry L, Brown Timothy T, Hagler Jr Donald J, Akshoomoff Natacha, Bartsch Hauke, Newman Erik, Thompson Wesley K, Bloss Cinnamon S, Murray Sarah S, Schork Nicholas, Kennedy David N, Kuperman Joshua M, McCabe Connor, Chung Yoonho, Libiger Ondrej, Maddox Melanie, Casey B J, Chang Linda, Ernst Thomas M, Frazier Jean A, Gruen Jeffrey R, Sowell Elizabeth R, Kenet Tal, Kaufmann Walter E, Mostofsky Stewart, Amaral David G, Dale Anders M, Pediatric Imaging Neurocognition, Genetics Study. The Pediatric Imaging, Neurocognition, and Genetics (PING) Data Repository. Neuroimage. 2016; 124: 1149–1154.

14. Kini Lohith G, Davis Kathryn A, Wagenaar Joost B. Data integration: Combined imaging and electrophysiology data in the cloud. Neuroimage. 2016; 124: 1175–1181.

15. Wang Lei, Alpert Kathryn I, Calhoun Vince D, Cobia Derin J, Keator David B, King Margaret D, Kogan Alex,r, L,is Drew, Tallis Marcelo, Turner Matthew D, Potkin Steven G, Turner Jessica A, Ambite Jose Luis. SchizConnect: Mediating neuroimaging databases on schizophrenia and related disorders for large-scale integration. Neuroimage. 2016; 124: 1155–1167.

16. Gorgolewski Krzysztof J, Varoquaux Gael, Rivera Gabriel, Schwarz Yannick, Ghosh Satrajit S, Maumet Camille, Sochat Vanessa V, Nichols Thomas E, Poldrack Russell A, Poline Jean-Baptiste, Yarkoni Tal, Margulies Daniel S. NeuroVault.org: a web-based repository for collecting and sharing unthresholded statistical maps of the human brain. Front Neuroinform. 2015; 9.

17. Gutman David A, Dunn Jr William D, Cobb Jake, Stoner Richard M, Kalpathy-Cramer Jayashree, Erickson Bradley. Web based tools for visualizing imaging data and development of XNATView, a zero footprint image viewer. Front Neuroinform. 2014; 8: 53.

18. Gao James S, Huth Alex,er G, Lescroart Mark D, Gallant Jack L. Pycortex: an interactive surface visualizer for fMRI. Front Neuroinform. 2015; 9.

19. Research Imaging Institute – Mango. http://ric.uthscsa.edu/mango/index.html. Accessed August 17, 2016.

20. NIDM API – nidm 1.0 documentation. http://nidm-api.readthedocs.org/en/latest/.

21. Wikipedia contributors. JSON. 2015. https://en.wikipedia.org/w/index.php?title=JSON&oldid=692109528. Accessed August 17, 2016.

22. Welcome to Flask – Flask Documentation (0.10). http://flask.pocoo.org/docs/0.10/.

23. Flask-RESTful – Flask-RESTful 0.2.1 documentation. http://flask-restful-cn.readthedocs.org/en/0.3.4/. Accessed August 17, 2016.

24. The Web framework for perfectionists with deadlines | Django. https://www.djangoproject.com/. Accessed August 17, 2016.

25. AWS Python Developer Center. https://aws.amazon.com/python/. Accessed August 17, 2016.

26. Google. Python Runtime Environment. https://cloud.google.com/appengine/docs/python/. Accessed August 17, 2016.

27. NIDM-Results 1.1.0. http://nidm.nidash.org/specs/nidm-results_110.html. Accessed August 17, 2016.

28. Jenkinson Mark, Beckmann Christian F, Behrens Timothy E J, Woolrich Mark W, Smith Stephen M. FSL. Neuroimage. 2012; 62: 782–790.

29. Neo4j, the World’s Leading Graph Database. http://neo4j.com/. Accessed August 17, 2016.

## A22 NeuroView: a customizable browser-base utility

### Anibal Sólon Heinsfeld, Alexandre Rosa Franco, Augusto Buchweitz, Felipe Meneguzzi

#### PUCRS, Porto Alegre, Rio Grande do Sul, Brazil

##### **Correspondence:** Anibal Sólon Heinsfeld (anibalsolon@gmail.com) – PUCRS, Porto Alegre, Rio Grande do Sul, Brazil

**Introduction**

The amount of data acquired for an fMRI experiment dimension wise is very large and a challenge for neuroscience studies, in particular for data analysis and visualization. Diverse tools have been developed to confront these challenges, but their analytical results can differ. Addressing those differences is not facilitated by existing tools. The goal of this Brainhack project was to build a flexible utility to analyze fMRI experimental results. This utility is called NeuroView. NeuroView allows researchers to extend the visualizations to their context: every visual behavior or interactions of this tool is customizable. We implemented NeuroView to work in Web-browsers, using JavaScript and the libraries D3.js and jQuery.

**Results**

We created three tools using NeuroView to best analyze our research results: CC200 search, SVM coefficients and Connectivity matrix. Each tool is used to aid the analysis of results in Machine Learning tasks. Each of these tools is described below in detail.

**CC200 search**

In this tool, we allow the user to find atlas regions (e.g. Left Putamen from Harvard-Oxford subcortical structural atlas) mapped to a specific parcellation. As as initial approach, the CC200 [1] parcellation method was used, since our analysis uses data from functional MRI. Since we parcellate our data into CC200’s ROIs in most of our studies, the identification of atlas regions became necessary to compare with results found in the literature. The search can be performed in two manners: it is possible to search for an atlas region (e.g. Putamen) and retrieve which parcels are included in this region, and it is possible to click an ROI in NeuroView to retrieve which atlas regions include the specific parcel.

**SVM coefficients**

**Fig. 19 (abstract A22).**
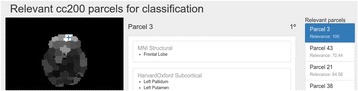
SVM coefficients tool showing the most relevant feature in a classification task

For the second tool, we created a user interface to identify the ROIs that contribute to the classification in a Support Vector Machine. The classification method uses task-based fMRI features to identify good and poor readers [2] Given a list of most relevant features, as shown in Fig. 19, we can show the features’ parcel in NeuroView and identify to which atlas regions this parcel belongs to.

**Connectivity matrix**

**Fig. 20 (abstract A22).**
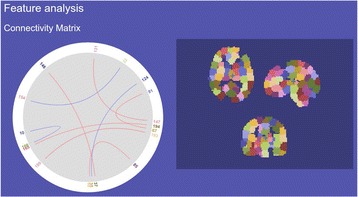
Deep learning

In the third study case, NeuroView was customized to interact with a connectivity chord plot (or connectogram) [3] This plot contains each CC200 parcel and chords that represent the connectivity between these parcels. Since we use the connectivity matrix as features for our deep learning method, we need to check which feature (i.e. the correlation between two parcels) most contributes to the classification. After thresholding 17995 features, we retrieve ten features that are more relevant in our analysis, as shown in Fig. 20. In the chord plot, a red chord indicates that two regions are correlated, and a blue chord indicates that two regions are anti-correlated. By clicking a chord, NeuroView highlights the regions that are connected by this chord. Thus, highlighted regions are correlated (or anti-correlated) given the chord color.

**Conclusion**

This is an initial version of a browser-based neuroimage viewer. The main focus is to develop an embeddable viewer, instead of a standalone desktop software. By doing so, research results can be presented on interactive views, enriching their analysis and interpretation. In our case study, NeuroView facilitates quick evaluation of features for machine learning algorithms, and promotes discussion about them, since the results will inform researchers about their data.

In future work, we aim to directly load Nifti images at client-side and support some AFNI features, such as voxel clustering.

**Availability of supporting data**

More information about this project can be found at: https://github.com/lsa-pucrs/neuroview

**Competing interests**

None.

**Author’s contributions**

ASH wrote the software, and ASH, FM, ARF, and AB wrote the report.

**Acknowledgements**

Report from 2015 Brainhack Americas (MX). The authors would like to thank the organizers and attendees of Brainhack MX and the developers of AFNI.

**References**

1. Craddock RC, James GA. A whole brain fMRI atlas generated via spatially constrained spectral clustering. Human brain mapping. 2012; 33.

2. Salles J, Piccolo LR, Zamo RS, Toazza R. Normas de desempenho em tarefa de leitura de palavras/pseudopalavras isoladas (LPI) para crian,cas de 1o ano a 7o ano. Estud e Pesqui em Psicol. 2013; 13: 397–419.

3. Irimia A, Chambers MC, Torgerson CM, Van Horn JD. Circular Representation of Human Cortical Networks for Subject and Population-level Connectomic Visualization. Neuroimage. 2012; 60: 1340–51.

## A23 DIPY: Brain tissue classification

### Julio E. Villalon-Reina^1^, Eleftherios Garyfallidis^2^

#### ^1^Imaging Genetics Center, USC Stevens Neuroimaging and Informatics Institute, Keck School of Medicine of USC, University of Southern California, Marina del Rey, California, USA; ^2^Département d'informatique, Université de Sherbrooke, Sherbrooke, Québec, Canada

##### **Correspondence:** Julio E. Villalon-Reina (julio.villalon@ini.usc.edu) – Imaging Genetics Center, USC Stevens Neuroimaging and Informatics Institute, Keck School of Medicine of USC, University of Southern California, Marina del Rey, California, USA

**Introduction**

DMRI is used for creating visual representations of the structural connectivity of the brain, also known as tractography. Research has shown that using a tissue classifier can be of great benefit to create more accurate representations of the underlying connections [1]

The aim of this project was to implement an image segmentation algorithm in DIPY [2] for classifying the different tissue types of the brain using structural T1 weighted images (T1-w) and diffusion MRI images (dMRI), and to incorporate the resulting tissue probability maps for Anatomically-Constrained Tractography (ACT) [3] We used Diffusion Power Maps (DPMs), which are scalar maps that are calculated from dMRI data and have a tissue contrast similar to the T1-w. By performing the tissue classification on dMRI derived scalar maps, the T1-w to dMRI registration step can be avoided.

**Approach**

We used a Bayesian approach for the segmentation in a similar fashion than the methods proposed in [4] and [5] by applying the Maximum-A-Posteriori (MAP) procedure. The prior probability was modeled with Markov Random Fields (MRF). The MRF distribution was modeled as a Gibbs distribution. We used the Expectation Maximization (EM) algorithm to update the tissue labels at each site and to update the parameters of the log-likelihood in all iterations.

**Results**

**Fig. 21 (abstract A23).**
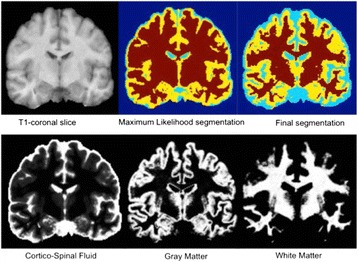
Example segmentations on T1 images

**Fig. 22 (abstract A23).**
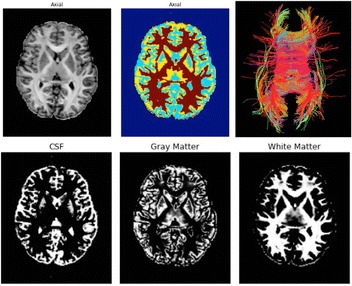
Example segmentations on Difussion Power Maps (DPM)

The first row of Fig. 21 shows the tissue classification on T1-w, the initial segmentation based on maximum likelihood and the final segmentation after 10 iterations and beta=0.1. Beta determines the weight of the neighborhood in the MRF model. These two parameters were tuned and validated by permuting 42 different combinations and calculating the Jaccard index between the segmentation of the proposed method against manually segmented brains from the IBSR dataset [http://www.nitrc.org/projects/ibsr]. The second row of Fig. 21 shows the probability maps of the three main tissue classes of the brain. The top row of Fig. 22 shows on the left the Diffusion Power Map (DPM), followed by its tissue classification and the streamlines from the corpus callosum reconstructed with ACT. The bottom row of Fig. 22 shows the tissue probability maps of the segmentation performed on a DPM.

**Conclusions**

We developed a segmentation algorithm based on a Bayesian framework by using the MAP-MRF approach and EM. The algorithm was tested on T1-w as well as on DPMs [6] The tissue specific probability maps from both the T1-w and the DPMs were then used for ACT. We were able to successfully run ACT with the tissue probability maps derived from the DPMs.

**Availability of Supporting Data**

More information about this project can be found at: https://github.com/villalonreina/dipy/tree/pve

**Competing interests**

None.

**Author’s contributions**

Julio E. Villalon-Reina and Eleftherios Garyfallidis performed the project and wrote the report.

**Acknowledgements**

The authors would like to thank the organizers and attendees of the 2015 OHBM Hackathon. Julio E. Villalon-Reina was funded by Google Summer of Code 2015.

**References**

1. Girard G, Whittingstall K, Deriche R, Descoteaux M. Towards quantitative connectivity analysis: reducing tractography biases. Neuroimage. 2014; 98: 266–278.

2. Garyfallidis Eleftherios, Brett Matthew, Amirbekian Bagrat, Rokem Ariel, Van Der Walt Stefan, Descoteaux Maxime, Nimmo-Smith Ian. Dipy, a library for the analysis of diffusion MRI data. Frontiers in Neuroinformatics. 2014; 8.

3. Robert ESmith, Jacques-Donald Tournier, Fern,o Calamante, Alan Connelly. Anatomically-constrained tractography: Improved diffusion streamlines tractography through effective use of anatomical information. NeuroImage. 2012; 62: 1924–1938.

4. Zhang Y, Brady M, Smith S. Segmentation of brain MR images through a hidden Markov random field model and the expectation-maximization algorithm. IEEE Trans Med Imaging. 2001; 20: 45–57.

5. Avants BB, Tustison NJ, Wu J, Cook PA, Gee JC. An open source multivariate framework for n-tissue segmentation with evaluation on public data. Neuroinformatics. 2011; 9: 381–400.

6. Flavio Dell'Acqua, Luis Lacerda, Marco Catani, Andrew Simmons. Anisotropic Power Maps: A diffusion contrast to reveal low anisotropy tissues from HARDI data. In: Proceedings Joint Annual Meeting ISMRM-ESMRMB, ISMRM2014, Milan, 2014. p. 0730.

